# Single‐Cell Transcriptomics Unravels Growth Factor Erv1‐Like Mediated Ferroptosis as a Key Driver of Intestinal Epithelial Dysfunction in Ulcerative Colitis

**DOI:** 10.1002/advs.202502014

**Published:** 2025-10-16

**Authors:** Ya Song, Fangyan Tan, Qian Song, Xiaohui Liao, Zhechuan Mei, Lin Lv

**Affiliations:** ^1^ Department of Gastroenterology The Second Affiliated Hospital of Chongqing Medical University 74 Linjiang Road, Yuzhong Chongqing 400010 China; ^2^ Department of Cardiology The Second Affiliated Hospital of Chongqing Medical University 74 Linjiang Road, Yuzhong Chongqing 400010 China; ^3^ The Second College of Clinical Medicine Chongqing Medical University 1 Yixue Road, Yuzhong Chongqing 400010 China; ^4^ Department of Nephrology The Second Affiliated Hospital Chongqing Medical University Chongqing 400010 China; ^5^ Kuanren Laboratory of Translational Lipidology Centre for Lipid Research Second Affiliated Hospital of Chongqing Medical University Chongqing 401336 China

**Keywords:** ferroptosis, inflammatory bowel disease, mitochondria, single‐cell RNA sequencing, ulcerative colitis

## Abstract

Inflammatory bowel disease (IBD) is a group of chronic, relapsing, and idiopathic inflammatory disorders of the gastrointestinal tract, primarily including ulcerative colitis (UC) and Crohn's disease (CD). The pathogenesis of UC remains incompletely understood, particularly regarding epithelial iron homeostasis and the regulation of cell death. In recent years, the application of single‐cell RNA sequencing (scRNA‐seq) has provided a powerful tool for dissecting cell‐type‐specific molecular mechanisms in UC. In this study, a comprehensive analysis of scRNA‐seq data revealed that *GFER* expression is significantly downregulated in intestinal epithelial cells of UC patients, suggesting a potential role in disease development. This finding is further validated in both a DSS‐induced colitis mouse model and an LPS‐induced in vitro inflammation model of intestinal epithelial cells, where *GFER* overexpression markedly inhibits the expression of ferroptosis‐related markers and alleviates inflammatory damage. What's more, it is found that GFER interacts with the iron‐regulating factor PCBP1 to help maintain intracellular iron homeostasis and may also reduce lipid peroxidation by activating the PGC‐1α/PPARγ signaling pathway, thereby inhibiting ferroptosis. This study is the first to demonstrate the critical role of GFER in regulating ferroptosis in UC, providing new insights into the pathogenesis of UC and identifying a potential therapeutic target for future intervention strategies.

## Graphical abstract

Single‐cell transcriptomic profiling of ulcerative colitis (UC) tissues identified downregulation of *GFER* in intestinal epithelial cells. Mechanistically, GFER interacts with PCBP1 to help maintain intracellular iron homeostasis and may also reduce lipid peroxidation by activating the PGC‐1α/PPARγ signaling pathway, thereby inhibiting ferroptosis. These findings highlight GFER as a critical regulator of UC pathogenesis and a promising therapeutic target for UC.

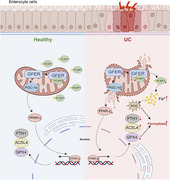

## 1 Introduction

Inflammatory bowel disease (IBD) is a group of chronic, recurrent, and idiopathic inflammatory disorders of the gastrointestinal tract, primarily comprising two subtypes: ulcerative colitis (UC) and Crohn's disease (CD).^[^ ^1^ ^]^ It is a condition resulting from the combined effects of genetic, environmental, and immune factors, posing a significant global public health challenge.

The pathogenesis of UC involves genetic susceptibility, immune dysregulation, and gut microbiota imbalance, among other complex factors.^[^ ^2^ ^]^ However, the precise molecular mechanisms remain incompletely understood. In recent years, with the development of single‐cell RNA sequencing (scRNA‐seq), researchers have been able to uncover disease‐specific gene expression patterns in different cellular subpopulations at single‐cell resolution. The study found that many risk genes associated with UC are cell type‐specific^[^ ^3^ ^]^ and different cell types may exhibit distinct responses to TNF‐α therapy in UC.^[^ ^4^ ^]^ ScRNA‐seq technology will provide deeper insights into the pathological processes of UC.

Ferroptosis, a novel form of regulated cell death, has garnered widespread attention in the context of UC.^[^ ^5^ ^]^ Characterized by iron‐dependent lipid peroxidation, ferroptosis not only disrupts the intestinal epithelial barrier but also exacerbates inflammation by releasing inflammatory mediators. Previous studies have shown that ferroptosis contributes to the progression of UC, highlighting its potential as a novel therapeutic target. Mitochondria play a critical regulatory role in ferroptosis, serving as essential hubs for intracellular iron metabolism and promoting lipid peroxidation through the generation of reactive oxygen species (ROS).^[^ ^6^ ^]^ Mitochondrial dysfunction is considered a key driver of ferroptosis in UC, though relatively little knowledge is available related to its specific molecular mechanisms.

The growth factor erv1‐like (*GFER*) gene encodes a sulfhydryl oxidase enzyme, also known as augmenter of liver regeneration (*ALR*). It is involved in mitochondrial protein folding and redox balance, closely linked to mitochondrial homeostasis and cell survival. Studies suggest that GFER may influence the occurrence of ferroptosis by regulating mitochondrial function and antioxidant defense mechanisms, thereby it may play a crucial role in UC pathogenesis. However, there have been few studies of the expression patterns of GFER in different cellular subpopulations of UC and its regulatory mechanisms.

Herein, leveraging scRNA‐seq technology, aim to investigate the expression patterns of the *GFER* gene in UC and its potential role in ferroptosis. The findings will elucidate the critical role of GFER in the pathological mechanisms of UC and provide novel perspectives and strategies for targeted therapies.

## 2 Result

### 2.1 ScRNA‐seq Reveals Transcriptional and Cellular Heterogeneity in UC

**Figure 1:**
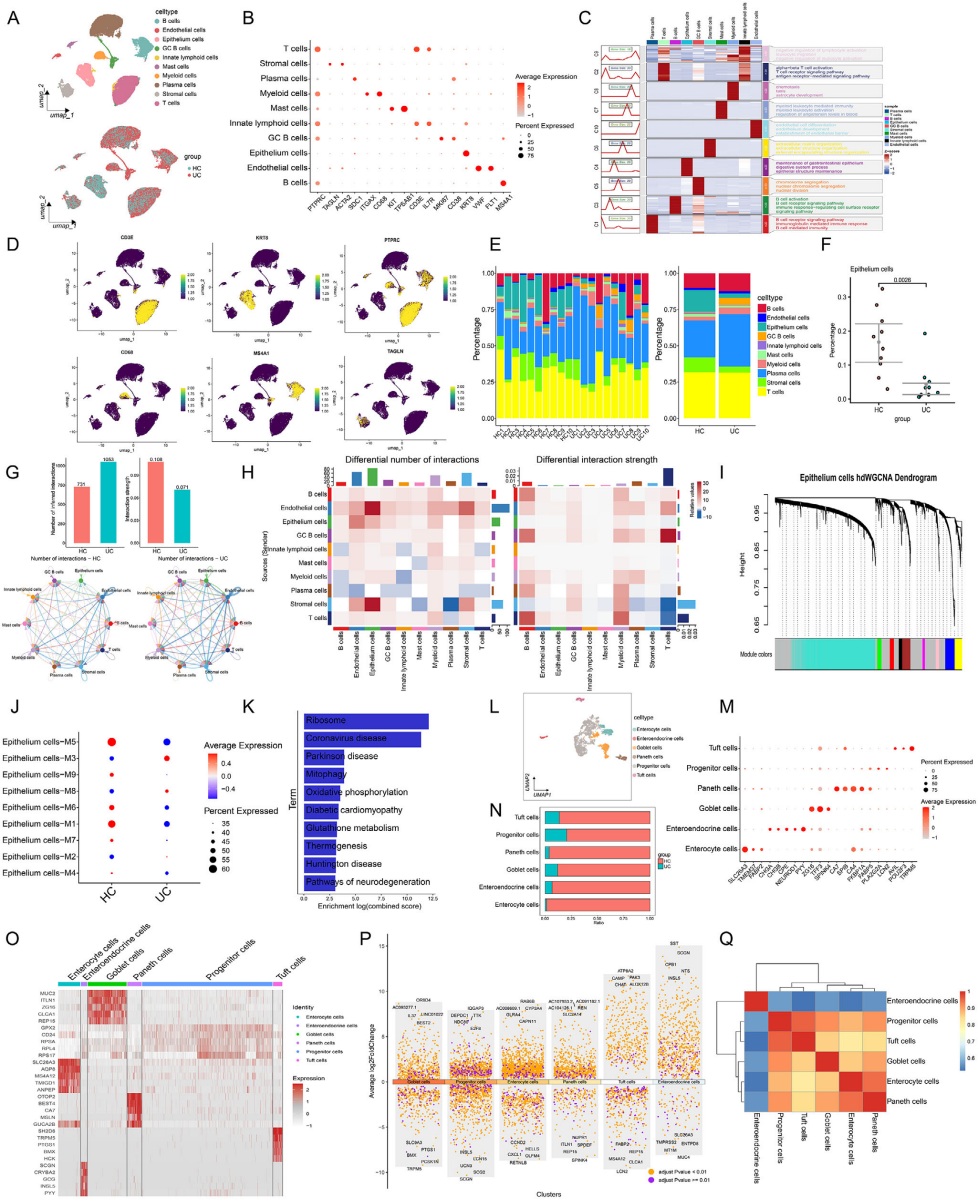
ScRNA‐seq reveals transcriptional and cellular heterogeneity in UC. A) 10 cell types identified by UMAP plot. B) Dot plot of the average expression of selected marker genes in 10 cell clusters. C) Heatmap of GO enrichment analysis in 10 cell clusters. D) UMAP plot of the marker genes in 10 cell clusters. E) Bar plots represented the proportion of cell clusters. F) Gene count plot of epithelium cells. G) Bar plot showing the number (left) or strength (right) of interactions in the cell–cell communication network from the CellChat analysis, circle plot showing the interaction number across all cell clusters. H) Heatmap showing the differences in the number (left) or strength (right) of interactions in the cell–cell communication network. I) Co‐expression network construction using the optimal soft‐thresholding power of 8, dividing the genes into 9 modules, and generating a dendrogram. J) Dot plot showing module enrichment across different cell types. K) KEGG enrichment analysis of the M5 module in colonic epithelial cells. L) 6 cell subclusters identified by UMAP plot. M) Dot plot of the average expression of selected marker genes in 6 cell subclusters. N) Bar plots represented the proportion of cell subclusters. O) The heatmap shows the expression profiles of representative marker genes in 6 cell subclusters. P) The volcano plot shows the DEGs of 6 cell clusters. Q) Correlation analysis of the 6 cell subpopulations.

To delineate the dynamic changes in the proportions and functions of various cell types during the progression of UC, we analyzed scRNA‐seq data from human clinical samples obtained from the GEO database. Based on the expression levels of marker genes, all cells were classified into 17 clusters and annotated into 10 cell types using the marker genes *PTPRC*, *SDC1*, *MS4A1*, *CD3E*, *IL7R*, *KRT8*, *ITGAX*, *CD68*, *MKI67*, *CD38*, *TAGLN*, *ACTA2*, *KIT*, *TPSAB1*, *VWF*, *FLT1*. These cell types included T cells, B cells, plasma cells, myeloid cells, mast cells, innate lymphoid cells, GC B cells, epithelial cells, endothelial cells, and stromal cells (**Figure** **1**A). The validity of our cell type classification was confirmed by the expression patterns of established cell type‐specific markers (Figure 1B,D) and the functional descriptions of these markers for each identified cell type (Figure 1C). Regarding changes in cellular composition during UC, we observed an increase in the number of myeloid cells and GC B cells (Figure 1E), indicating that chronic inflammatory responses lead to a reorganization of the cellular environment. Concurrently, there was a reduction in the number of epithelial and stromal cells in the colonic microenvironment (Figure 1E), particularly in intestinal epithelial cells (Figure 1F). In parallel, we applied a linear mixed‐effects model (LMM) to evaluate the association between the relative proportions of each cell cluster and disease status, incorporating batch, cohort, age, and sex as covariates for adjustment. As shown in Figure S1 (Supporting Information), these covariates had no significant impact on epithelial cells. This observation underscores that in UC, toxins and enzymes released by certain gut microbiota (such as lipopolysaccharides and proteases) can directly damage epithelial and stromal cells. Pro‐inflammatory factors and oxidative stress also promote apoptosis in these cells, and the disruption of barrier function exacerbates the inflammatory response, creating a vicious cycle that further reduces the number of epithelial cells.

The maintenance of morphology and function in multicellular organisms largely depends on intercellular communication. Therefore, using the ligand‐receptor interaction tool CellChat, we systematically inferred changes in the communication network among single cells in UC. Surprisingly, compared to the HC group, the UC group showed an increased number of interactions but a decreased interaction strength (Figure 1G). The colonic epithelial cell cluster in UC had more interactions with other cells, but the interaction strength was significantly weaker (Figure 1H). Subsequent hdWGCNA analysis of the colonic epithelial cell population divided them into 9 modules (Figure 1I). Analysis of the characteristics of each module revealed that module 5 showed significant differences between the HC and UC groups (Figure 1J). KEGG enrichment analysis of module 5 indicated that it was primarily enriched in pathways related to mitophagy, oxidative phosphorylation, and glutathione metabolism (Figure 1K). Further subclustering of the epithelial cell population based on marker genes *SLC26A3*, *TMEM37*, *FABP2*, *CHGA*, *CHGB*, *CPE*, *NEUROD1*, *PYY*, *ZG16*, *TFF3*, *SPINK4*, *CA7*, *SPIB*, *CA4*, *FKBP1A*, *FABP5*, *PLA2G2A*, *LCN2*, *AVIL*, *POU2F3*, *TRPM*, resulted in 6 cell subpopulations: Enterocyte cells, Enteroendocrine cells, Goblet cells, Paneth cells, Progenitor cells, and Tuft cells (Figure 1L,M). Compared to the HC group, the UC group showed a significant reduction in the proportion of each cell subpopulation, with enterocyte cells showing the most pronounced change (Figure 1N). Figure 1O–Q show the expression profiles of representative marker genes in different intestinal epithelial cell subclusters, the distribution of DEGs across subclusters, and the correlations among the cell subclusters. Previous studies have reported that enterocyte cells, which constitute the majority of intestinal epithelial absorptive cells in the colonic epithelial cell population, play a significant role.^[^ ^19^, ^20^, ^21^ ^]^

### 2.2 In UC, Enterocyte Cells are Primarily Associated with Ferroptosis

**Figure 2:**
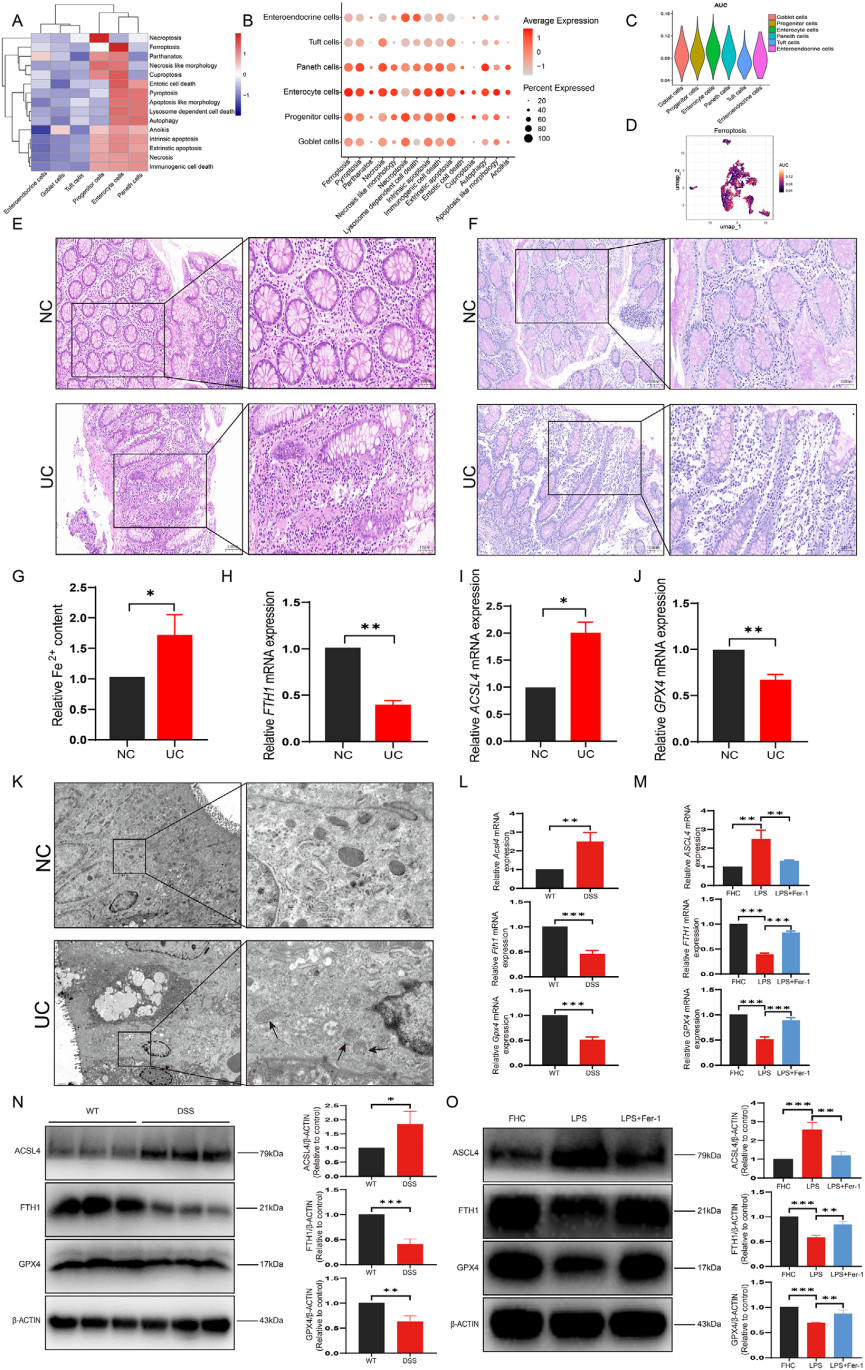
In UC, enterocyte cells are primarily associated with ferroptosis. A) Heatmap of GSVA scores for 15 modes of cell death in colonic epithelial cells. B) Dot plot of AddModuleScore for 15 modes of cell death in colonic epithelial cells. C) The violin plot of the AUCell score for ferroptosis in colonic epithelial cells. D) The UMAP plot of the AUCell score for ferroptosis in colonic epithelial cells. E) The H&E staining of the clinical tissues. F) The PAS staining of the clinical tissues. G) Ferrous iron content of clinical tissues. H) Relative mRNA expression of *FTH1* in clinical tissues. I) Relative mRNA expression of *ACSL4* in clinical tissues. J) Relative mRNA expression of *GPX4* in clinical tissues. K) Transmission electron micrographs of the clinical tissues. L) Relative mRNA expression levels of *Acsl4*, *Fth1*, and *Gpx4* from animal tissues. M) Relative mRNA expression levels of *ACSL4*, *FTH1*, and *GPX4* in FHC cells after Fer‐1 administration. N) Protein expression levels of ACSL4, FTH1, and GPX4 from animal tissues. O) Protein expression levels of ACSL4, FTH1, and GPX4 in FHC cells after Fer‐1 administration.

To explore the primary modes of cell death in each cell subpopulation, we used GSVA, Addmodulescore, and AUCell to score the epithelial cell population. We found that enterocyte cells were mainly associated with ferroptosis (**Figure** **2**A–D). We collected clinical specimens and detected their clinicopathological phenotypes. The UC group exhibited crypt structural disorganization and inflammatory cell infiltration compared to the NC group (Figure 2E–F), increased iron content (Figure 2G), and PCR results showed increased mRNA expression of *ACSL4* but decreased mRNA expression of *GPX4* and *FTH1* (Figure 2H–J). Electron microscopy results showed that the intestinal mucosal epithelial cells in the UC group exhibited slight edema, signs of ferroptosis, disordered and sparse microvilli, large areas of atrophy and shedding, slightly widened intercellular spaces, and mitochondria that appeared slightly shrunken, mostly smaller and rounder, with high electron density in the inner matrix and reduced or disappeared cristae. In contrast, the intestinal mucosal epithelial cells in the control group showed relatively normal structure, intact cell membranes, evenly distributed organelles in the cytoplasm, no significant swelling, and a relatively intact intestinal barrier structure (Figure 2K). In a DSS‐induced colitis mouse model, the DSS group showed higher expression of ACSL4 and lower expression of GPX4 and FTH1 compared to the WT group (Figure 2L,N). Ferrostatin‐1 (Fer‐1), a ferroptosis inhibitor, was used in an LPS‐induced inflammation model of colonic epithelial cells.^[^ ^22^ ^]^ Compared to the normal LPS group, the expression of ACSL4 was decreased in the LPS+Fer‐1 group, while the expression of GPX4 and FTH1 was increased (Figure 2M,O). These results indicate that ferroptosis occurs in colonic epithelial cells in UC.

### 2.3 GFER is Downregulated in UC

**Figure 3:**
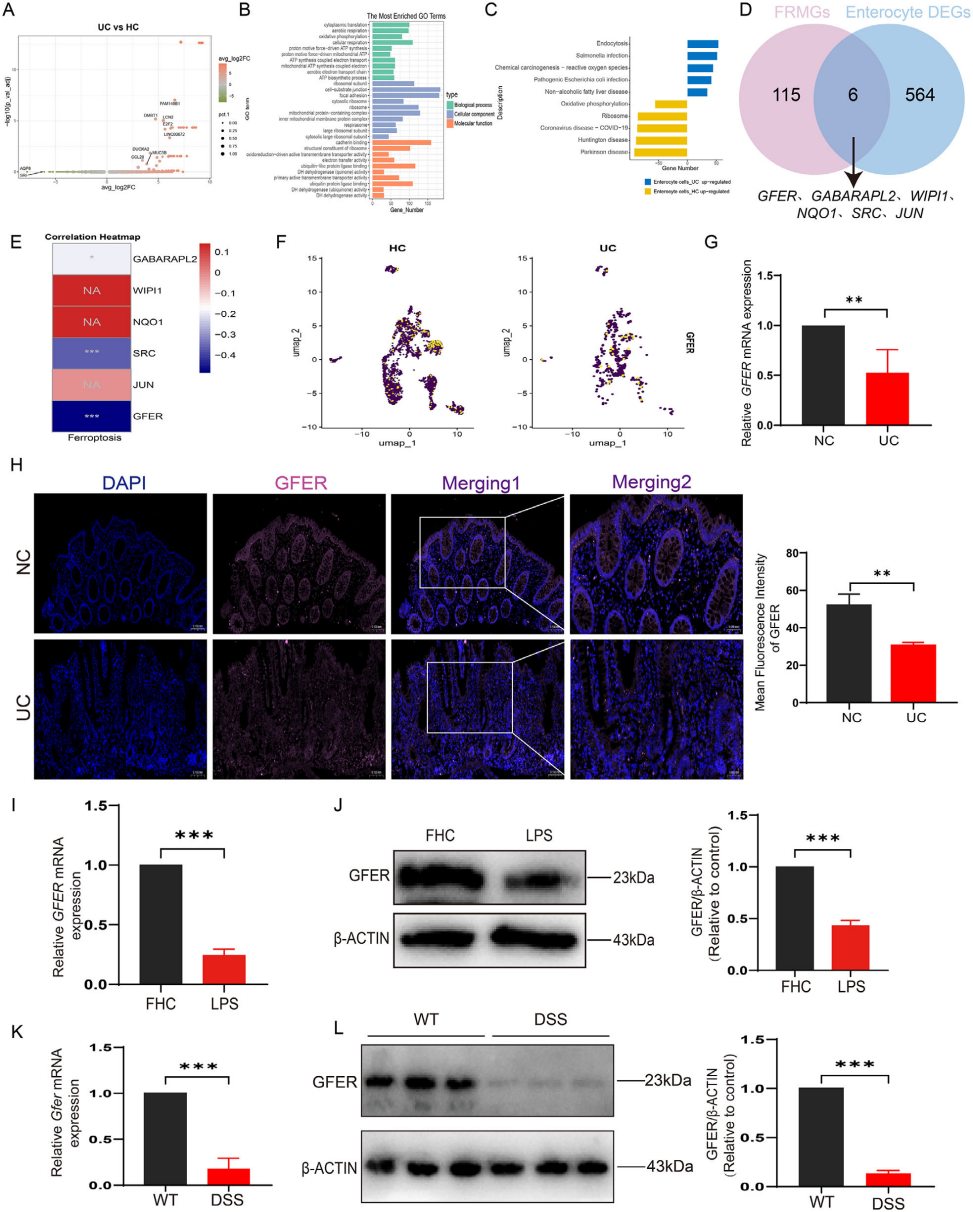
GFER is downregulated in UC. A) Volcano plot of DEGs in the enterocyte cells. B) GO enrichment analysis of DEGs in the enterocyte cells. C) KEGG enrichment analysis of DEGs in the enterocyte cells. D) Wayne diagram. E) Correlation analysis of six genes with ferroptosis at the transcriptome level. F) The UMAP plot of the GFER of the enterocyte cells. G) Relative mRNA expression of *GFER* in clinical tissues. H) Immunofluorescence plots of GFER from clinical tissues. I) Relative mRNA expression of *GFER* in FHC cells. J) Protein expression level of GFER in FHC cells. K) Relative mRNA expression of *Gfer* from animal tissues. L)Protein expression levels of GFER from animal tissues.

Enterocyte cells‐specific differential gene analysis between UC and HC was presented in the volcano plot (**Figure** **3**A). GO enrichment analysis showed that the DEGs in enterocyte cells were primarily enriched in processes such as ATP biosynthetic process, aerobic electron transport chain, mitochondrial ATP synthesis coupled electron transport, proton motive force‐driven mitochondrial ATP synthesis, cellular respiration, oxidative phosphorylation, aerobic respiration, mitochondrial protein‐containing complex, inner mitochondrial membrane protein complex, respirasome, oxidoreduction‐driven active transmembrane transporter activity, and electron transfer activity (Figure 3B). KEGG enrichment analysis of the upregulated and downregulated pathways revealed enrichment in Chemical carcinogenesis‐reactive oxygen species, and Oxidative phosphorylation pathways (Figure 3C). Mitochondria, often referred to as the “powerhouses” of the cell, also regulate apoptosis and serve as cellular factories for energy production, calcium homeostasis, and iron metabolism,^[^ ^23^ ^]^ mitochondria are also the main source of intracellular ROS generation, and they are also involved in ROS clearance.^[^ ^24^, ^25^ ^]^ Enterocyte cells, closely associated with mitochondrial function, play a significant role in UC progression. Mitochondria regulate ferroptosis by influencing iron metabolism, lipid peroxidation, redox balance, and energy metabolism, which affect its occurrence and progression. Dysfunctional mitochondria and ferroptosis interactions may exacerbate cell damage and are critical mechanisms in various diseases. Protective interventions targeting mitochondria could offer novel therapeutic approaches for ferroptosis‐related conditions. Using the GeneCards database and PubMed we identified 121 FRMGs. Intersecting these with DEGs in enterocyte cells, 6 FRMGs related to UC progression were identified: *GABARAPL2*, *WIPI1*, *NQO1*, *SRC*, *JUN*, and *GFER* (Figure 3D). To determine their consistency at the bulk transcriptome level, we assessed correlations of the six genes with ferroptosis in UC samples from the GSE75214 dataset.^[^ ^26^ ^]^ Only *GABARAPL2*, *SRC*, and *GFER* showed significant differences, with *GFER* exhibiting the most pronounced change (Figure 3E). UMAP mapping of scRNA‐seq data showed that *GFER* was specifically expressed in the enterocyte cells (Figure 3F). Clinical sample analysis confirmed that *GFER* mRNA expression was significantly lower in UC patients compared to normal controls (Figure 3G). Immunofluorescence indicated that GFER was primarily expressed in colonic epithelial crypts, with fluorescence intensity changes consistent with PCR results (Figure 3H). In an LPS‐induced colonic epithelial cell model, GFER expression was significantly downregulated compared to the FHC group (Figure 3I,J). Similarly, in a DSS‐induced colitis mouse model, GFER expression was markedly reduced in the DSS group compared to the WT group (Figure 3K,L).

To investigate the effect of TNF‐α treatment, we analyzed scRNA‐seq data from the GSE282122 dataset. This publicly available dataset includes samples from patients with UC (*n* = 22), CD (*n* = 16), and healthy controls, featuring longitudinal samples collected before and after treatment, along with detailed clinical metadata. Using the same marker genes, epithelial cells were classified into six subpopulations (Figure S2A,B, Supporting Information). Figure S2C,D (Supporting Information) show that the proportion of enterocyte cells fluctuates significantly across different pathological states—including healthy, Pre/Post‐Remission, and Pre/Post‐Non‐Remission—suggesting that this cell type may play an important role in the progression of UC. Compared to the healthy group, the ferroptosis score is elevated in UC. Additionally, in the remission group, the ferroptosis score before TNF‐α treatment is higher than after treatment. Regardless of remission status, the ferroptosis score is significantly increased under inflamed conditions, suggesting that ferroptosis may be involved in the persistent inflammatory response observed in UC (Figure S2E, Supporting Information). *GFER* expression is markedly downregulated in UC compared to healthy controls. Irrespective of remission status, *GFER* expression levels are consistently lower in inflamed tissues than in non‐inflamed tissues (Figure S2F, Supporting Information), indicating a strong association between *GFER* expression and the inflammatory state. Notably, no statistically significant differences in *GFER* expression were observed before and after TNF‐α treatment, regardless of remission status (Figure S2F, Supporting Information). This lack of significance may be attributed to a limited sample size, which could be addressed in future studies with larger cohorts. Alternatively, the heterogeneity of inflamed tissue sampling may contribute to inter‐individual variability, potentially masking the true expression dynamics of *GFER* in response to treatment. Figure S2G,H (Supporting Information) illustrates the distribution of ferroptosis scores and *GFER* expression across various cell types. The results suggest that enterocytes may be the primary cell type responsible for both *GFER* expression and ferroptosis occurrence. Figure S2I (Supporting Information) reveals a significant negative correlation between *GFER* expression and ferroptosis score (Spearman ρ = –0.509, P < 2.2e‐16), suggesting that *GFER* may function as a negative regulator of ferroptosis. Figure S2J,K (Supporting Information) employs Monocle‐based pseudotime analysis to further elucidate the dynamic changes in *GFER* expression, ferroptosis‐related gene sets, and inflammation scores during remission and non‐remission phases of UC. The results show that both ferroptosis and inflammation scores increase during the early stage of disease in the remission phase and decline after treatment, implying that ferroptosis may be associated with the therapeutic effects of TNF‐α. However, based on our current analysis, the relationship between TNF‐α treatment and *GFER* expression remains unclear and warrants further investigation through additional experimental validation.

### 2.4 GFER Protects Against Ferroptosis in Colonic Epithelial Cells in an Inflammation Model

**Figure 4:**
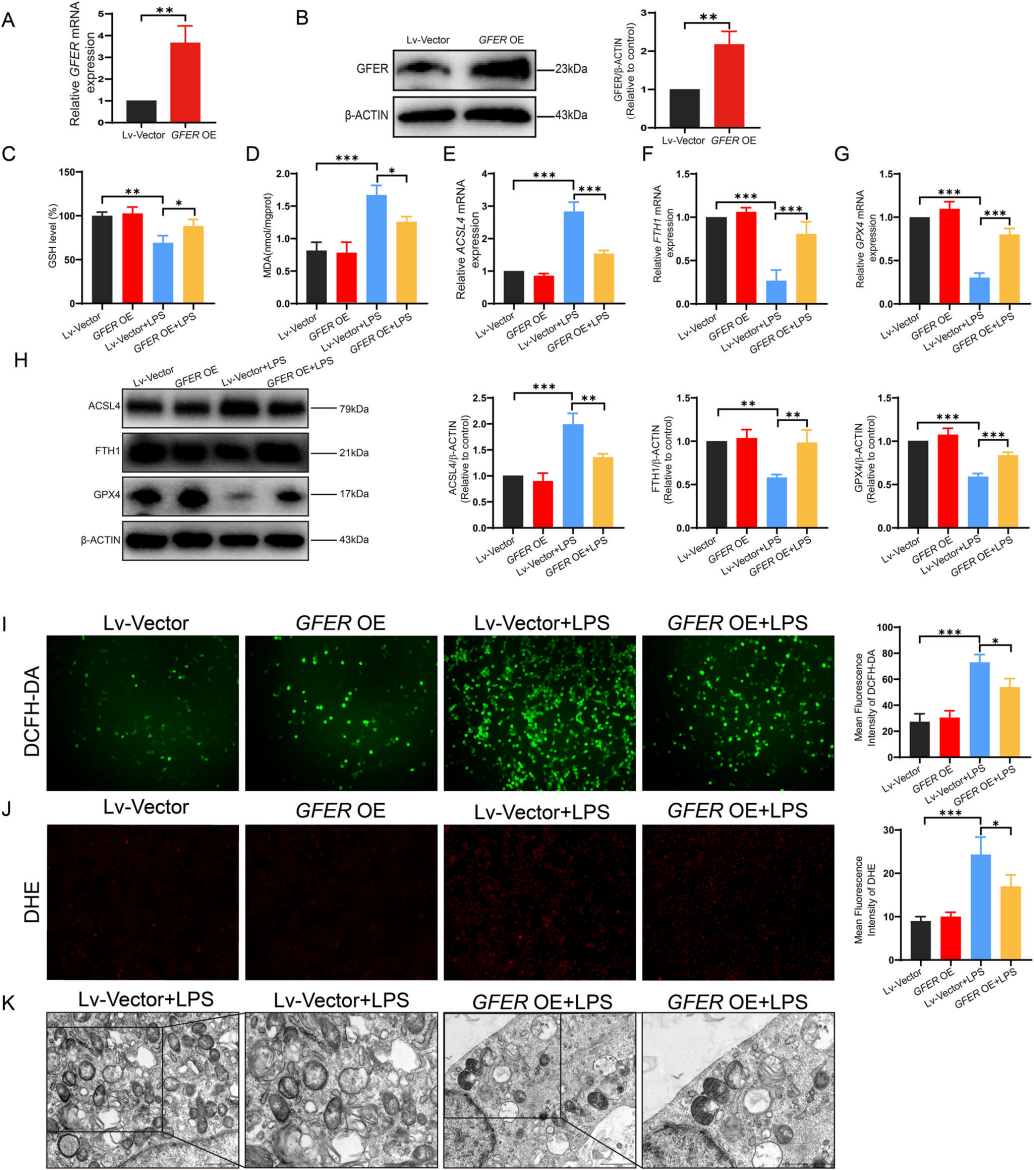
GFER protects against ferroptosis in colonic epithelial cells in an inflammation model. A) Relative mRNA expression level of *GFER* in FHC cells after *GFER* overexpression. B) Protein expression level of GFER in FHC cells after *GFER* overexpression. C) GSH content in FHC cells after *GFER* overexpression. D) MDA content in FHC cells after *GFER* overexpression. E) Relative mRNA expression level of *ACSL4* in FHC cells after *GFER* overexpression. F) Relative mRNA expression level of *FTH1* in FHC cells after *GFER* overexpression. G) Relative mRNA expression level of *GPX4* in FHC cells after *GFER* overexpression. H) Protein expression levels of ACSL4, FTH1, and GPX4 in FHC cells after *GFER* overexpression. I) ROS content in FHC cells after *GFER* overexpression. J) Superoxide anion content in FHC cells after *GFER* overexpression. K) Transmission electron microscopy images of FHC cells after *GFER* overexpression.

To further investigate the role of GFER in UC, we overexpressed *GFER* in FHC colonic epithelial cells using lentiviral transfection and assessed its effect on ferroptosis. PCR and western blot confirmed successful overexpression of *GFER* in FHC cells (**Figure** **4**A,B). Ferroptosis markers, including GSH and MDA, were measured. Compared to the CON+LPS group, the *GFER* OE+LPS group showed significantly increased GSH levels (Figure 4C), while MDA levels significantly decreased (Figure 4D).

We also assessed the ferroptosis‐related markers. Overexpression of *GFER* significantly reduced ACSL4 mRNA and protein levels (Figure 4E,H), while GPX4 and FTH1 mRNA and protein levels were significantly increased (Figure 4F,G,H). As ROS levels and mitochondrial damage are hallmarks of ferroptosis, we evaluated mitochondrial ROS levels and ultrastructural changes in the inflammatory intestinal cell model. Intracellular ROS levels were measured using the DCFH‐DA fluorescent probe method.^[^ ^27^ ^]^ Therefore, we tested the ROS content of *GFER* after overexpression, and found that the fluorescence intensity of DCFH‐DA was significantly decreased in the *GFER* OE+LPS group compared with the CON+LPS group (Figure 4I). DHE is one of the most commonly used superoxide anion fluorescence detection probes, which can effectively detect ROS.^[^ ^28^ ^]^ Compared with the CON+LPS group, the fluorescence intensity of DHE in the *GFER* OE+LPS group was significantly reduced (Figure 4J). The above results showed that *GFER* overexpression inhibited the ROS production induced by LPS in colonic epithelial cells. In addition, TEM showed loss of mitochondrial cristae, mitochondrial membrane rupture, and abnormal mitochondrial morphology in the CON+LPS group, while partially reversed by the *GFER* OE+LPS group (Figure 4K). Subsequently, FHC cells were transfected with Si*GFER* and treated with the ferroptosis inhibitor Fer‐1. We observed a decrease in ACSL4 expression and an increase in FTH1 and GPX4 expression (Figure S3, Supporting Information), indicating that Fer‐1 can mitigate the enhanced ferroptosis induced by *GFER* silencing.

### 2.5 GFER Protects Against Ferroptosis in Animal Experimental Colitis Models

**Figure 5:**
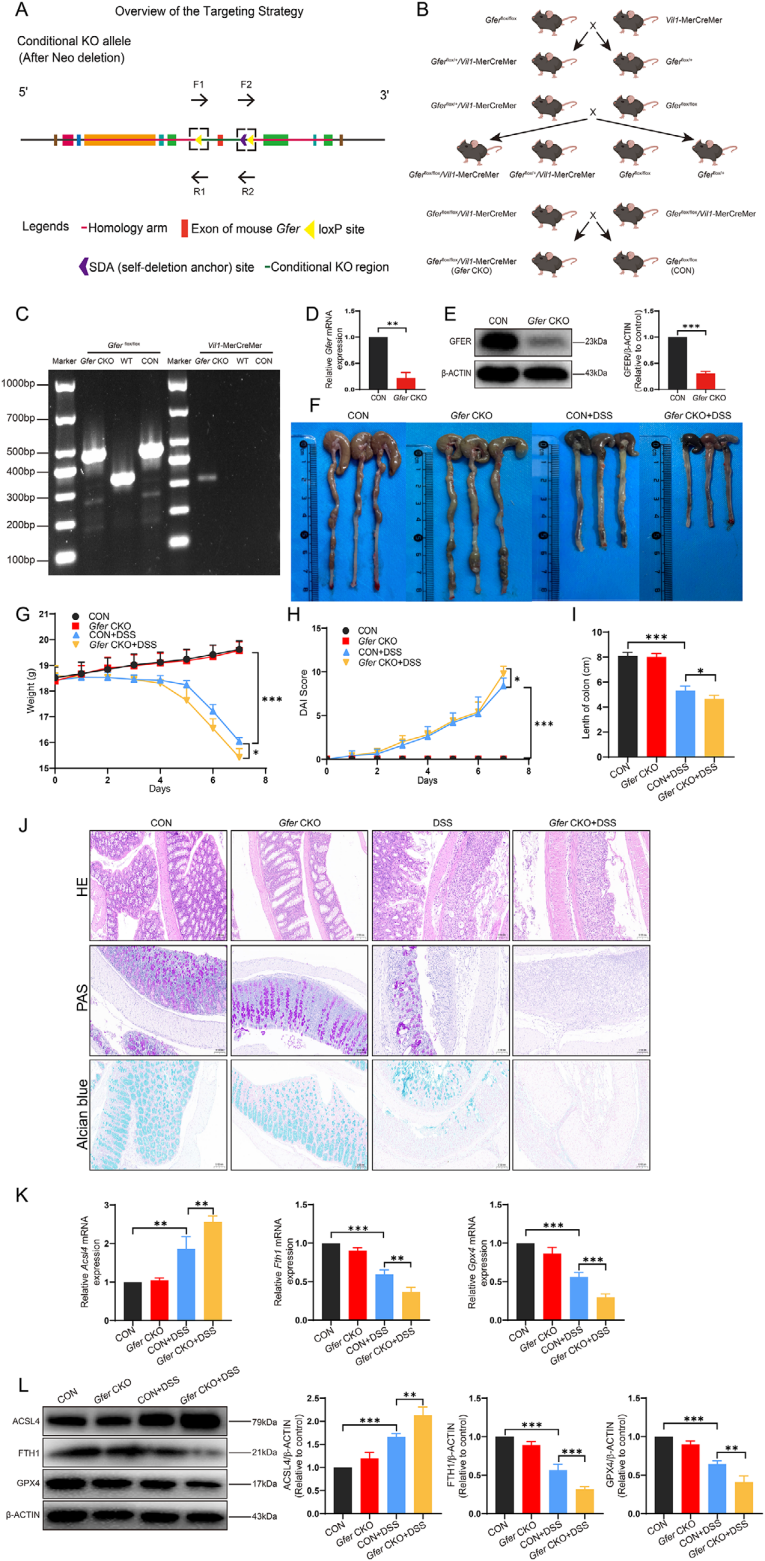
In animal models, *Gfer* knockout induces the occurrence of ferroptosis. A) Schematic diagram of the knockout strategy. B) Schematic diagram of the breeding strategy. C) Agarose gel electrophoresis for genotyping of mice. D) Relative mRNA expression level of *Gfer* in colonic crypts after *Gfer* knockout. E) Protein expression level of GFER in colonic crypts after *Gfer* knockout. F) Gross morphology of the colon in mice after *Gfer* knockout. G) Body weight changes in mice after *Gfer* knockout. H) DAI score in mice after *Gfer* knockout. I) Colon length in mice after *Gfer* knockout. J) H&E, PAS, and Alcian blue staining of the intestinal tract in mice after *Gfer* knockout. K) Relative mRNA expression levels of *Acsl4*, *Fth1*, and *Gpx4* in colonic crypts after *Gfer* knockout. L) Protein expression levels of ACSL4, FTH1, and GPX4 in colonic crypts after *Gfer* knockout.

To explore the role of GFER in colitis in vivo, we constructed *Gfer* CKO mice (**Figure** **5**A). The breeding strategy is shown in Figure 5B. The genotype of the mice was identified by agarose gel electrophoresis of DNA samples from the mice, as shown in Figure 5C, successfully obtaining mice with the genotype *Gfer* ^flox/flox^, *Vil1*‐MerCreMer. Intestinal crypts were extracted, and PCR and western blot were used to determine the mRNA and protein expression levels of GFER, showing that the *Gfer* gene had been knocked down in the intestinal crypts (Figure 5D,E). A 3.0% DSS feeding model was used to construct a colitis mouse model. After one week, it was found that specific knockout of the *Gfer* gene in the colonic epithelium resulted in lighter body weight (Figure 5G), higher DAI scores (Figure 5H), and shorter colon lengths (Figure 5F,I) in the *Gfer* CKO+DSS group compared to the CON+DSS group. Histopathology of the colon showed varying degrees of colonic ulcers, mucosal edema, goblet cell loss, crypt swelling and destruction, and varying degrees of inflammatory cell infiltration in the mucosa and submucosa, with epithelial cell damage (Figure 5J). Extraction of colon tissue crypts and detection of mRNA content revealed that compared to the DSS group, the *Gfer* CKO+DSS group had higher expression of *Acsl4* and lower expression of *Gpx4* and *Fth1* (Figure 5K). Similarly, the *Gfer* CKO+DSS group had higher protein expression of ACSL4 and lower protein expression of GPX4 and FTH1 (Figure 5L), indicating that GFER deficiency can partially exacerbate the ferroptosis phenotype. In addition, we administered AAV‐mediated *Gfer* overexpression in mice and found that colonic tissues from *Gfer*‐overexpressing mice exhibited decreased ACSL4 expression and increased FTH1 and GPX4 expression (Figure S4, Supporting Information). These results indicate that *Gfer* may inhibit ferroptosis in mice with experimental colitis.

### 2.6 GFER Regulates Iron Metabolism by Direct Binding to PCBP1 during UC Ferroptosis

**Figure 6:**
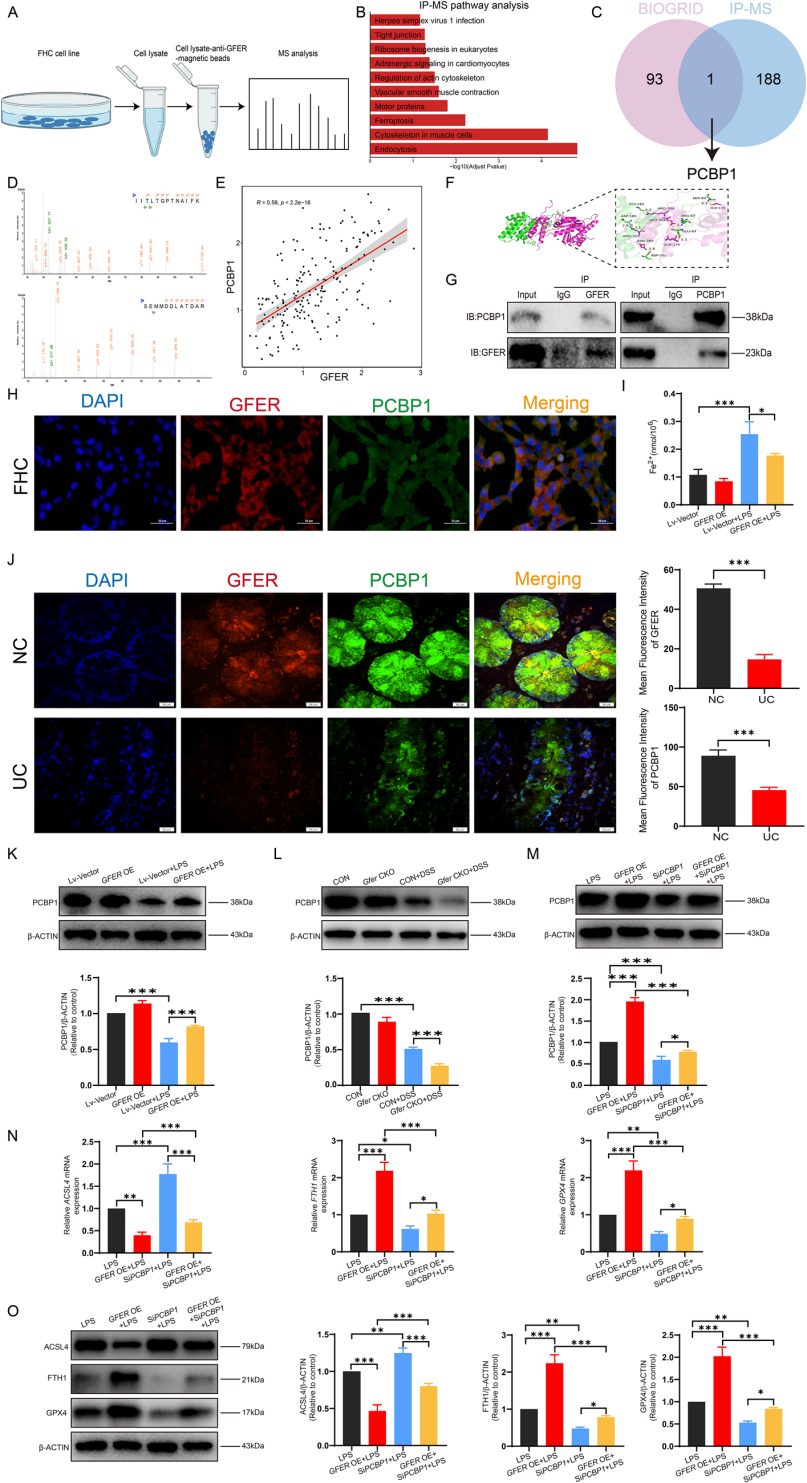
GFER regulates iron metabolism by direct binding to PCBP1 during UC ferroptosis. A) IP‐MS process flowchart. B) Bar graph of the top 10 KEGG pathway enrichment terms for proteins interacting with GFER. C) Wayne diagram. D) Mass spectrometry identification of PCBP1 and GFER. E) Spearman correlation analysis of *PCBP1* and *GFER* in enterocyte cells. F) Molecular docking diagram of GFER and PCBP1. G) Co‐IP results of GFER and PCBP1. H) Co‐localization immunofluorescence of GFER and PCBP1 in FHC cells. I) Ferrous ion content in FHC cells after *GFER* overexpression. J) Immunofluorescence images of GFER and PCBP1 in clinical samples. K) Protein expression level of PCBP1 in FHC cells after *GFER* overexpression. L) Protein expression level of PCBP1 in colonic crypts after *Gfer* knockout. M) Protein expression level of PCBP1 in FHC cells after *GFER* overexpression and Si*PCBP1* treatment. N) Relative mRNA expression levels of *ACSL4*, *FTH1*, and *GPX4* in FHC cells after *GFER* overexpression and Si*PCBP1* treatment. O) Protein expression levels of ACSL4, FTH1, and GPX4 in FHC cells after *GFER* overexpression and Si*PCBP1* treatment.

In search for binding partners of GFER in the colonic epithelium, we searched for GFER‐associated binding partners (**Figure** **6**A) by IP‐MS experiments in human colonic epithelial cells, found that 189 proteins associated with GFER, then we performed these 189 proteins to KEGG enrichment analysis, and the top10 entries by adjust P‐value ranking could be enriched to Endocytosis, Ferroptosis, Ribosome biogenesis in eukaryotes, and so on (Figure 6B). Subsequently, to increase the feasibility of the experiment, we collected 94 proteins in the BIOGRID database that may interact with GFER, taking the intersection with 189 IP‐MS data, found only PCBP1 (Figure 6C). The present findings suggest that PCBP1 may be a key downstream target of GFER in colitis. MS identification of PCBP1 and GFER was presented in Figure 6D, and Spearman correlation analysis of single cell transcriptome found a significant positive correlation between *GFER* and *PCBP1* in the enterocyte cell population (Figure 6E). Moreover, the molecular docking of GFER and PCBP1 revealed some binding sites (Figure 6F) between GFER and PCBP1. To further verify the binding relationship, Co‐IP was performed (Figure 6G), and immunofluorescence of FHC cells revealed that GFER colocalized (Figure 6H), the results indicate that there may be a positional interaction between GFER and PCBP1. Following *GFER* overexpression, Co‐IP demonstrated an increased interaction between GFER and PCBP1, whereas LPS stimulation reduced this interaction (Figure S5, Supporting Information). Detection of intracellular ferrous iron levels revealed that *GFER* overexpression led to a reduction in ferrous iron, while LPS stimulation resulted in increased levels (Figure 6I). Immunofluorescence experiments found low expression of GFER and PCBP1 in the colonic crypt part of the UC group (Figure 6J). Western blot measurements revealed that *GFER* overexpression increased PCBP1 expression (Figure 6K). Knockout of *Gfer* potentiated the inhibitory effect of DSS on PCBP1 (Figure 6L). These results suggest that the interaction between GFER and PCBP1 may inhibit PCBP1 degradation in the LPS‐induced cellular inflammation model. Furthermore, the effects of *GFER* overexpression against the enhancement of the ferroptosis markers GPX4 and FTH1 and the inhibition of the pro‐ferroptosis marker ACSL4 were rescued by silencing *PCBP1* (Figure 6M,N,O). These results suggest that GFER regulates ferroptosis in the colonic epithelium by targeting PCBP1 and the associated iron ion metabolism.

### 2.7 GFER Affects the GPX4‐Mediated Ferroptosis Process by Indirectly Regulating the PGC‐1α /PPAR‐γ Signaling Pathway in UC

**Figure 7:**
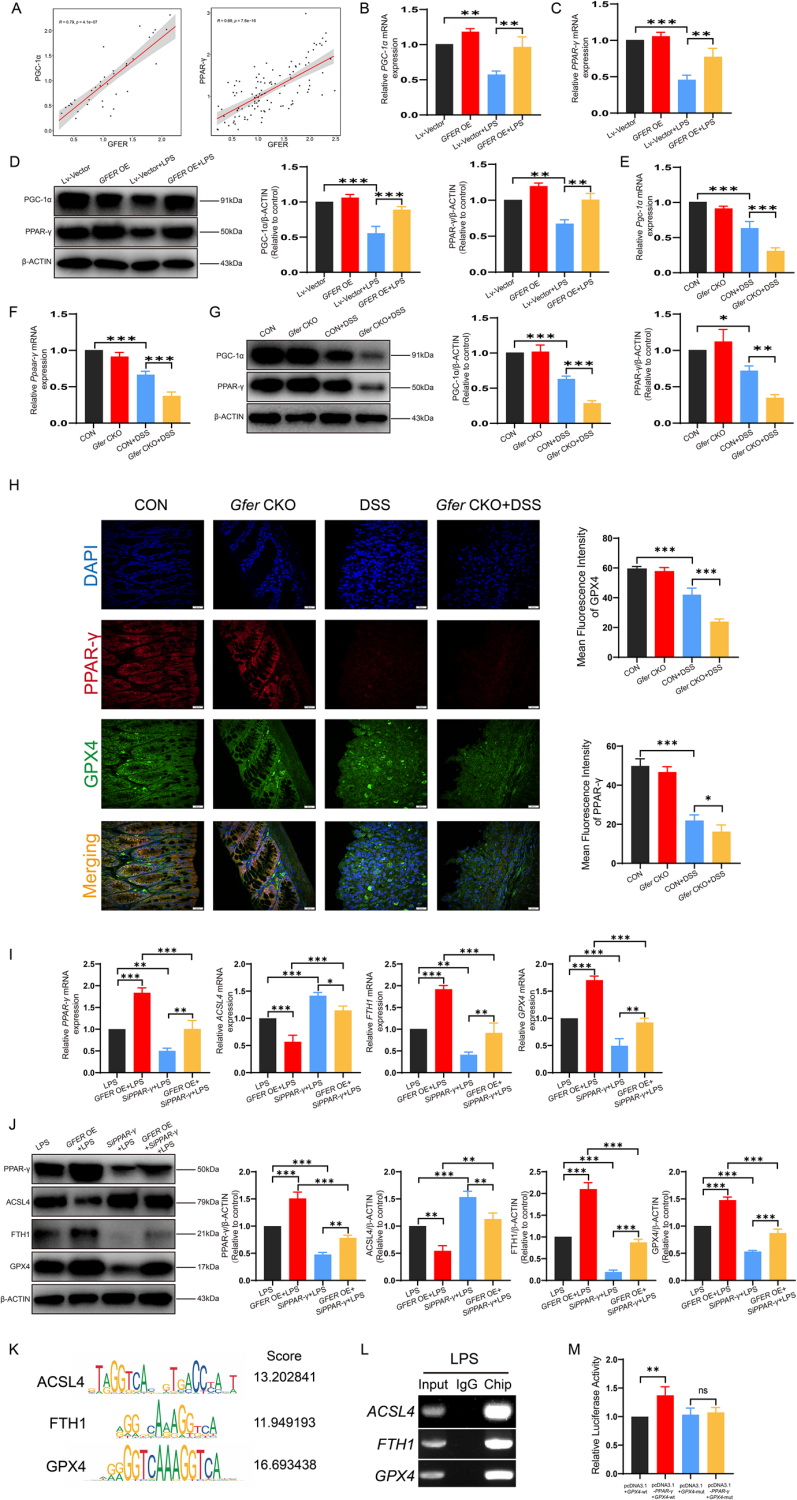
GFER affects the GPX4‐mediated ferroptosis process by indirectly regulating the PGC‐1α/PPAR‐γ signaling pathway in UC. A) Spearman correlation analysis of *GFER* with *PGC‐1α* and *PPAR‐γ* in enterocyte cells. B) Relative mRNA expression levels of *PGC‐1α* in FHC cells after *GFER* overexpression. C) Relative mRNA expression levels of *PPAR‐γ* in FHC cells after *GFER* overexpression. D) Protein expression levels of PGC‐1α and PPAR‐γ in FHC cells after *GFER* overexpression. E) Relative mRNA expression levels of *Pgc‐1α* in colonic crypts after *Gfer* knockout. F) Relative mRNA expression levels of *Ppar‐γ* in colonic crypts after *Gfer* knockout. G) Protein expression levels of PGC‐1α and PPAR‐γ in colonic crypts after *Gfer* knockout. H) Immunofluorescence images of PGC‐1α and PPAR‐γ in colonic tissues after *Gfer* knockout. I) Relative mRNA expression levels of *PPAR‐γ*, *ACSL4*, *GPX4*, *FTH1* in FHC cells after *GFER* overexpression and Si*PPAR‐γ* treatment. J) Protein expression levels of PPAR‐γ, ACSL4, GPX4, FTH1 in FHC cells after *GFER* overexpression and Si*PPAR‐γ* treatment. K) Jaspar database prediction of PPAR‐γ binding sites on the DNA of ACSL4, FTH1, and GPX4. L) ChIP results. M) Dual‐luciferase assay results.

Previous studies also found that GFER was significantly enriched for lipid metabolism signaling after GFER knockout and was associated with the PPAR signaling pathway.^[^ ^29^ ^]^ However, no protein related to the PPAR signaling pathway was found in our mass spectrometry results, so we speculated that GFER does not directly regulate the PPAR signaling pathway, but does indirectly regulates the PPAR signaling pathway. PGC‐1α is the master regulator of mitochondrial generation, which can activate a range of genes related to mitochondrial biosynthesis.^[^ ^30^, ^31^ ^]^ Previous studies have found that GFER, acting as a mitochondrial protein, can regulate PGC‐1α‐associated mitochondrial biogenesis.^[^ ^32^ ^]^ PGC‐1α enhances PPAR‐γ transcriptional activity and promotes the expression of downstream target genes.^[^ ^33^ ^]^ Therefore, we used Spearman correlation analysis on the correlation analysis of *PGC‐1α*/*PPAR‐γ*, genes related to the PPAR signaling pathway in enterocyte cells, and found that *GFER* showed a significant positive correlation with *PGC‐1α*/*PPAR‐γ* in the colonic epithelial cell population (**Figure** **7**A). A large number of previous studies have demonstrated that PPAR‐γ plays an important role in UC.^[^ ^34^, ^35^ ^]^ Currently, PPAR‐γ has been identified as a therapeutic target for various UC treatments, leading us to hypothesize that GFER may regulate ferroptosis through the PGC‐1α/PPAR‐γ pathway. PCR and western blot demonstrated that *GFER* overexpression increased PGC‐1α and PPAR‐γ expression in the LPS group (Figure 7B–D), whereas *Gfer* knockout decreased their expression in the DSS group (Figure 7E–G). Immunofluorescence staining showed that GPX4 and PPAR‐γ expression was reduced in the colonic crypts of the DSS group, and this reduction was further exacerbated by *Gfer* knockout (Figure 7H). Additionally, in the LPS‐induced inflammatory model of FHC cells, *GFER* overexpression and *PGC‐1α* silencing (Si*PGC‐1α*) were performed simultaneously. Western blot analysis showed that compared to the *GFER* OE+LPS group, the *GFER* OE+Si*PGC‐1α*+LPS group exhibited decreased expression of PPAR‐γ, FTH1, and GPX4, and increased expression of ACSL4 (Figure S6, Supporting Information). These results suggest that silencing *PGC‐1α* can rescue the effects of *GFER* overexpression on ferroptosis‐related markers. Furthermore, silencing *PPAR‐γ* rescued the effects of *GFER* overexpression on ferroptosis markers, including the enhancement of GPX4 and FTH1 and the suppression of ACSL4 (Figure 7I,J). As PPAR‐γ is a transcription factor, predictions from the JASPAR database suggested potential binding sites of PPAR‐γ within the promoter regions of ACSL4, GPX4, and FTH1 (Figure 7K). Using PPAR‐γ‐specific antibodies, a ChIP assay in LPS‐induced colonic epithelial cells confirmed potential binding, as validated by agarose gel electrophoresis of PCR products (Figure 7L). Since the PPAR‐γ binding site score was highest for GPX4 in the JASPAR database, we selected this site for mutation. A dual‐luciferase assay confirmed the potential PPAR‐γ binding site on the DNA of GPX4 (Figure 7M). These results suggest that GFER regulates ferroptosis in colonic epithelial cells through the PGC‐1α/PPAR‐γ signaling pathway.

### 2.8 GFER Inhibition Exacerbates Intestinal Barrier Damage in Colitis Animal Models

**Figure 8:**
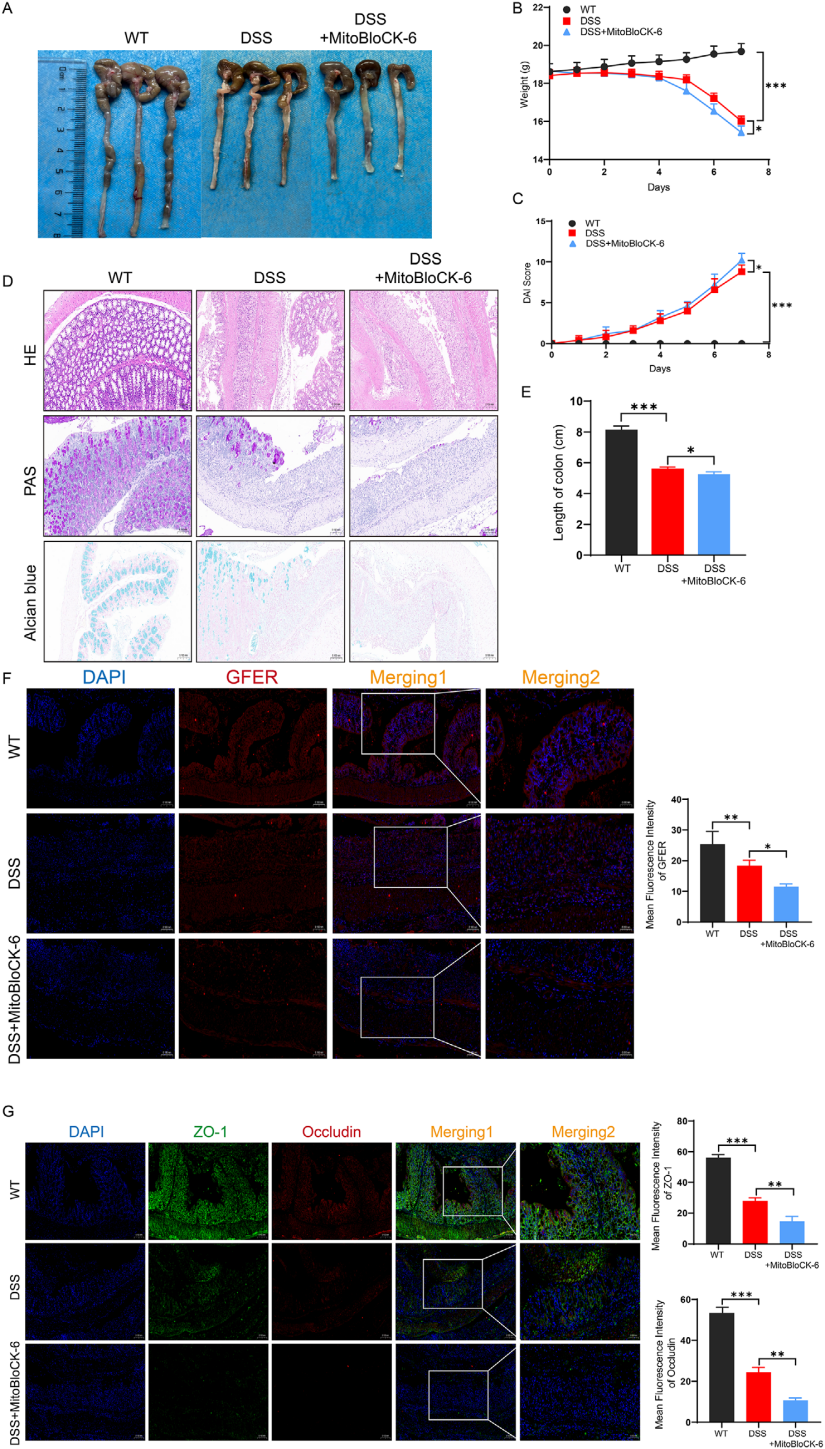
GFER inhibition exacerbates intestinal barrier damage in colitis animal models. A) Gross morphology of colonic tissues after MitoBloCK‐6 treatment. B) Body weight changes in mice after MitoBloCK‐6 treatment. C) DAI score in mice after MitoBloCK‐6 treatment. D) H&E, PAS, and Alcian blue staining of the intestinal tissues in mice after MitoBloCK‐6 treatment. E) Colon length in mice after MitoBloCK‐6 treatment. F) Immunofluorescence images of GFER in colonic tissues after MitoBloCK‐6 treatment. G) Immunofluorescence images of ZO‐1 and Occludin in colonic tissues after MitoBloCK‐6 treatment.

MitoBloCK‐6, an effective Erv1/ALR inhibitor.^[^ ^36^, ^37^ ^]^ We further investigated the effects of MitoBloCK‐6 on GFER and ferroptosis in DSS‐induced colitis animal models. In mice treated with MitoBloCK‐6, compared to the DSS group, body weight was significantly reduced (**Figure** **8**A,B), DAI scores were higher (Figure 8C), and colons were shorter (Figure 8E). H&E staining of colonic tissue revealed more severe pathological changes, including disrupted crypt structures and increased inflammatory infiltration (Figure 8D). Immunofluorescence of colonic tissue showed that GFER expression was significantly reduced in the MitoBloCK‐6+DSS group compared to the DSS group (Figure 8F). Additionally, intestinal barrier proteins ZO‐1 and Occludin were further decreased in the MitoBloCK‐6+DSS group (Figure 8G), indicating that MitoBloCK‐6 exacerbated the impairment of intestinal barrier integrity in DSS‐induced colitis.

## 3 Discussion

UC is a complex and chronic inflammatory condition primarily affecting the colon and rectum. Despite significant advances in understanding the pathogenesis of UC, there remains a critical need to develop more refined and targeted therapeutic strategies. With the advent of scRNA‐seq technologies, we now have unprecedented tools to explore the cellular and molecular complexity of UC. ScRNA‐seq enables the identification of cell type‐specific transcriptomic profiles and rare cellular subsets that may play pivotal roles in disease progression. Additionally, scRNA‐seq allows for the dissection of cellular interactions and signaling pathways at an unparalleled resolution, offering new insights into the microenvironmental dynamics within the inflamed tissue.

**Figure 9:**
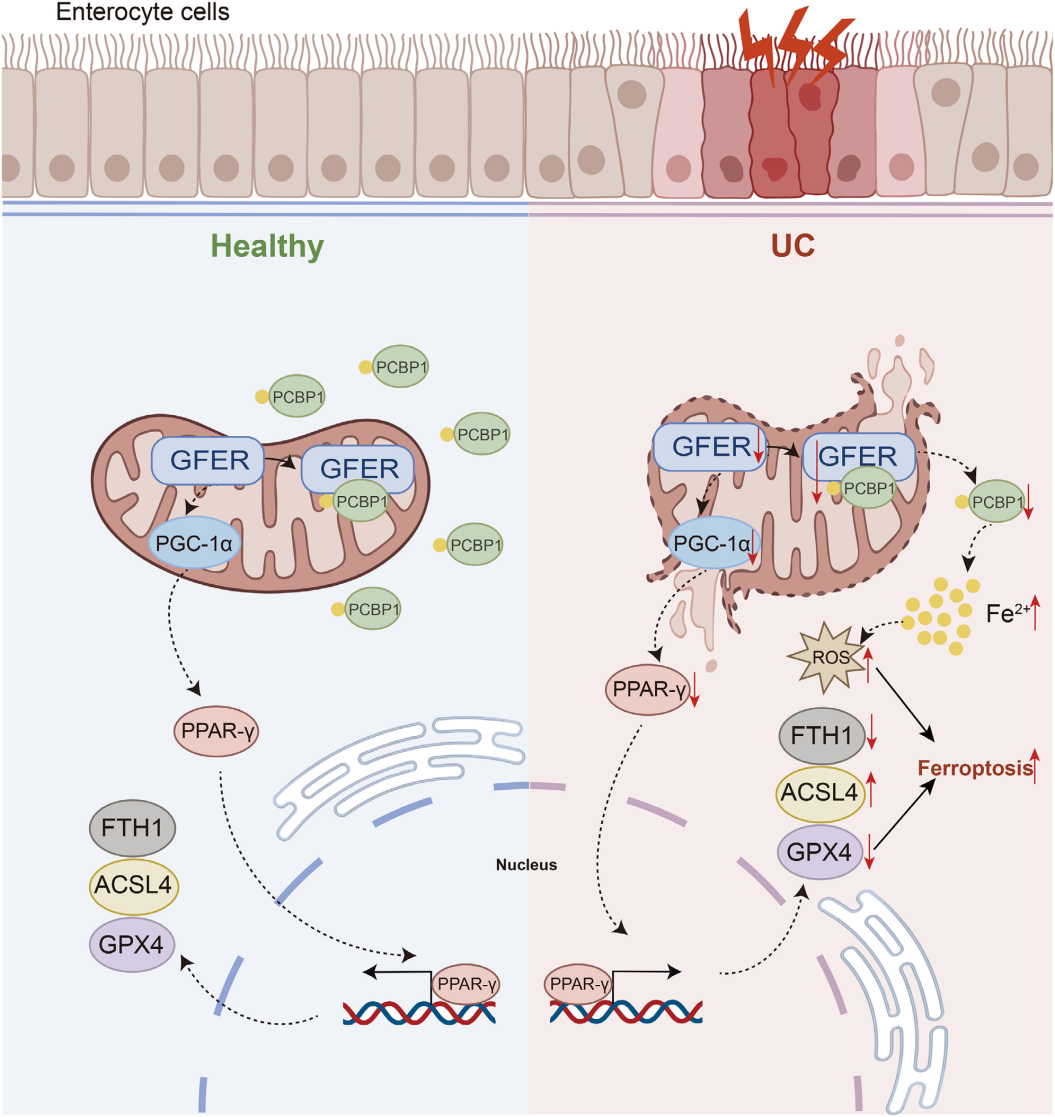
Schematic diagram illustrating the molecular mechanism.

These advancements can help pinpoint specific molecular targets and pathways involved in UC pathogenesis. Moreover, the ability to map changes at the single‐cell level between healthy and diseased states facilitates the identification of potential biomarkers for early diagnosis and therapeutic response monitoring. Overall, leveraging scRNA‐seq technologies provides a promising avenue for uncovering the intricate mechanisms underlying UC and developing precise, personalized treatments for this debilitating condition.^[^ ^38^ ^]^ By integrating scRNA‐seq data from the GEO database, we documented a significant increase in inflammatory response pathways within the human colonic microenvironment during UC. This reflects a strong immune counteraction to the inflamed tissue. Notably, we observed an increase in the number of myeloid cells and GC B cells, accompanied by a decrease in epithelial and stromal cells, further supporting the robust immune response against UC. Among these findings, the proportion of colonic epithelial cells was notably altered and closely associated with ferroptosis, a form of programmed cell death.

Ferroptosis is an iron‐dependent form of programmed cell death, primarily driven by the accumulation of lipid peroxidation and redox imbalance, ultimately leading to the destruction of cell membranes.^[^ ^39^, ^40^ ^]^ The main pathological features of UC are the disruption of the intestinal epithelial barrier and the excessive release of inflammatory factors.^[^ ^41^, ^42^ ^]^ The accumulation of lipid peroxides and the disruption of intestinal barrier function caused by ferroptosis exacerbate the pathological processes of UC.^[^ ^43^ ^]^ Reduced expression of GPX4 is closely associated with intestinal epithelial cell damage in UC patients.^[^ ^44^ ^]^ Ferroptosis, through lipid peroxidation, oxidative stress, and amplification of inflammatory signaling, plays a key role in colonic epithelial inflammation and may exacerbate the pathological processes of IBD. Enterocytes are one of the major cell types in the intestinal epithelium, located on the inner walls of the small intestine and colon. They play critical roles in intestinal barrier function, nutrient absorption, and immune regulation. Dysregulation of enterocyte cell function is closely associated with UC. Enterocyte cells are central to the pathogenesis and progression of colitis, and their dysfunction, including barrier disruption, activation of pro‐inflammatory signaling, and reduced regenerative capacity, exacerbates intestinal inflammation, leading to tissue damage and chronic pathological progression.^[^ ^45^, ^46^ ^]^ By protecting the enterocyte cells and modulating their associated mechanisms, it could provide effective strategies for the treatment of colitis.

The DEGs in enterocyte cells are significantly enriched in mitochondrial and reactive oxygen species‐related pathways, indicating that metabolic dysregulation in colonic epithelial cells involves ferroptosis‐associated mitochondrial dysfunction. Among ferroptosis‐related mitochondrial genes, GFER may play a pivotal role. Mitochondria are crucial for cellular iron metabolism, contributing to the synthesis of iron‐sulfur clusters and heme. Excess iron can promote lipid peroxidation through ROS generation, which is a central step in ferroptosis. Electrons leaking from the mitochondrial electron transport chain can react with excess H_2_O_2_ produced by iron, generating hydroxyl radicals that accelerate lipid peroxidation. Mitochondria are the primary source of ROS. During ferroptosis, elevated ROS levels oxidize unsaturated lipids to form lipid peroxides, leading to membrane damage and cell death. In this process, GSH depletion inhibits the activity of GPX4, a key enzyme responsible for detoxifying lipid peroxides. Mitochondrial dysfunction exacerbates this process, causing uncontrolled ROS levels. Metabolites from the TCA cycle, such as succinate and pyruvate, participate in mitochondrial metabolism and may influence intracellular iron and ROS levels, thus regulating ferroptosis sensitivity. Additionally, changes in mitochondrial membrane potential during ferroptosis affect the proton gradient, further influencing mitochondrial metabolic function and oxidative stress levels.

This study identifies GFER as a potential regulatory factor in UC, providing evidence that mitochondrial homeostasis plays a critical role in structural and metabolic remodeling in UC. GFER is a widely expressed multifunctional protein with roles in maintaining mitochondrial stability and regulating cellular metabolic pathways.^[^ ^47^ ^]^ The GFER protein exists in three isoforms: 23, 21, and 15 kDa. The longest form is located only in the mitochondria, and it plays a crucial role in the maturation of cellular solute Fe/S proteins and mitochondrial respiration in yeast.^[^ ^48^ ^]^ However, the role of GFER in UC remains unclear. Using scRNA‐seq, we found that *GFER* expression was significantly reduced in colonic epithelial cells in UC compared to normal tissues, and its expression showed a strong negative correlation with ferroptosis scores, suggesting that GFER may act as a negative regulator of ferroptosis. In addition, we described the expression of GFER in patients who achieved remission and non‐remission after TNF‐α treatment,^[^ ^49^ ^]^ although GFER expression did not show significant differences across remission status, this result may be attributed to the limited sample size or the heterogeneity of inflamed tissue regions. Moreover, the effect of TNF‐α treatment on GFER expression remains unclear, which opens a new avenue for exploring its potential as a therapeutic target. Previous studies have linked GFER to mitochondrial biogenesis and ferroptosis.^[^ ^29^, ^50^ ^]^ Consistent with our bioinformatics analysis, GFER was primarily expressed in colonic epithelial cells, and its expression was notably decreased in UC patients, potentially correlating with disease severity. Lorenzo Polimeno et al. reported that the mRNA expression levels of *NRF2* and *GFER* were significantly reduced in sporadic colorectal adenoma tissues compared to normal mucosa in the control group.^[^ ^51^ ^]^ Additionally, Li Guo et al. identified that GFER expression is associated with prognosis in colorectal adenocarcinoma, with higher GFER expression correlating with a survival advantage.^[^ ^52^ ^]^ Previous studies on GFER in cancer (such as colorectal adenocarcinoma and sporadic adenomas) have primarily focused on its association with prognosis and oxidative stress. However, its direct mechanistic role in the progression from UC to colorectal cancer remains unclear and warrants further investigation. Moving forward, we aim to collect clinical data to investigate whether GFER is involved in colitis‐to‐cancer transition and explore its correlation with prognosis.

The study demonstrates that the downregulation of GFER expression in colonic epithelial cells of UC disrupts ferroptosis and interferes with PCBP1‐mediated iron metabolism signaling. Mitochondria are central to intracellular iron metabolism, including the synthesis of iron‐sulfur clusters and heme production. Imbalances in iron metabolism lead to the accumulation of free iron, which facilitates the Fenton reaction, generating more ROS. These ROS induce lipid peroxidation, a hallmark of ferroptosis. Previous studies have shown that GFER alleviates mitochondrial dysfunction by promoting mitochondrial fusion, thereby mitigating organ damage, such as in kidney injury. This highlights the crucial role of GFER in maintaining mitochondrial homeostasis and regulating iron and ROS levels, which are integral to ferroptosis and its pathological implications in UC.^[^ ^53^ ^]^ There is also literature found that GFER can also alleviate the occurrence of development in kidney ferroptosis.^[^ ^54^, ^55^ ^]^ PCBP1, which is an important regulator of iron metabolism, is responsible for the transport of iron ions into iron storage proteins.^[^ ^56^ ^]^ Previous studies have shown that PCBP1 acts as an iron chaperone in ferroptosis and can regulate the expression of key ferroptosis‐related genes, including GPX4, ACSL4, and FTH1.^[^ ^57^, ^58^ ^]^ Iron metabolism is a critical step in the occurrence of ferroptosis. Our study found that GFER may interact with PCBP1 to regulate the expression of key ferroptosis markers. Specifically, when PCBP1 was knocked down, the expression of GPX4 and FTH1 in colonic epithelial cells decreased, while ACSL4 expression increased. Overexpression of *GFER* partially reversed the effects of *PCBP1* knockdown, indicating that GFER can modulate ferroptosis markers through PCBP1. In the LPS‐induced inflammatory model of FHC cells, the binding between GFER and PCBP1 was reduced, which may expose more ubiquitination enzyme binding sites on PCBP1, potentially leading to increased ubiquitination and decreased PCBP1 expression in the inflammatory context. Overexpression of *GFER*, by binding more PCBP1, can inhibit the LPS‐induced reduction in PCBP1 expression. We plan to conduct further experiments to investigate the impact of GFER on the ubiquitination of PCBP1 in greater detail.

Additionally, our findings reveal that GFER indirectly regulates ferroptosis in colonic epithelial cells by modulating the PGC‐1α/PPAR‐γ signaling pathway and associated lipid metabolism. PGC‐1α plays a vital role in mitochondrial biogenesis and oxidative metabolism, maintaining mitochondrial homeostasis and preventing excessive ROS production due to mitochondrial damage. Studies have shown that TFEB partially regulates ROS homeostasis through PGC‐1α, and defects in this pathway may play a pivotal role in colitis progression.^[^ ^59^ ^]^ PPAR‐γ is a critical anti‐inflammatory nuclear receptor that reduces the release of pro‐inflammatory cytokines by inhibiting the NF‐κB and MAPK signaling pathways. Activation of PPAR‐γ can alleviate local intestinal inflammation and protect intestinal epithelial cells. By preventing increased intestinal permeability, PPAR‐γ plays a protective role. Some PPAR‐γ agonists, such as thiazolidinediones, have been studied for their therapeutic potential in UC. PGC‐1α enhances the transcriptional activity of PPAR‐γ, and together they regulate antioxidant genes, inhibit ferroptosis, regulate mitochondrial function, and reduce the generation of iron‐dependent ROS, thereby lowering ferroptosis sensitivity. Both PGC‐1α and PPAR‐γ have significant protective roles in the regulation of ferroptosis. They cooperate to modulate lipid metabolism, mitochondrial function, and antioxidant defense, thereby reducing lipid peroxidation and ROS production, which suppresses ferroptosis.

In summary, by integrating scRNA‐seq and bulk transcriptomic data, we identified the protective role of the ferroptosis‐related mitochondrial gene *GFER* in enterocyte cells during colitis. Furthermore, GFER regulates the progression of ferroptosis in UC through PCBP1‐mediated iron metabolism and PGC‐1α/PPAR‐γ‐mediated lipid metabolism pathways (**Figure** **9**). These findings provide potential targets for the development of new therapeutic strategies for UC.

## 4 Experimental Section

### Obtained ScRNA‐seq Datasets and ScRNA‐seq Data Analysis

A PubMed search for scRNA‐seq studies on UC was first conducted. Based on study design, only scRNA‐seq studies of whole colonic tissues were included, excluding those that analyzed T cells or myeloid cells isolated by flow cytometry. Ultimately, 20 samples were incorporated from two studies (GSE231993 and GSE214695), comprising 10 UC samples and 10 healthy control (HC) samples, which were analyzed using the Seurat R package.^[^ ^7^ ^]^ For each sample, genes and feature counts were identified, and cells with fewer than 200 or more than 6000 features were filtered out. Cells with mitochondrial RNA percentages > 15% or ribosomal RNA percentages < 3% were also removed. DoubletFinder^[^ ^8^ ^]^ was used for doublet analysis with default settings for each sample, and all potential doublets were removed. Subsequently, the scRNA‐seq datasets were merged into a larger Seurat object. To integrate scRNA‐seq data from multiple samples and eliminate technical batch effects, the Harmony algorithm^[^ ^9^ ^]^ for data correction was applied. The Harmony integration was performed using the following key parameters: *θ* = 2, max.iter.harmony = 10, epsilon.harmony = 1e‐05, *λ* = 1, and reduction = “pca”. In this study, the default convergence parameters was retained to ensure stability and reproducibility, while specifically evaluating the effect of different levels of integration strength as controlled by the theta parameter. Visualization analysis revealed that the default setting (*θ* = 2) achieved effective batch mixing while preserving biological variability, and therefore, no further adjustments were made. After Harmony correction, cells from different batches overlapped extensively in the low‐dimensional space, and the batch‐driven separation was markedly reduced, indicating that the batch effects were effectively corrected. The integrated data underwent normalization, scaling, and processing for PCA analysis. Data visualization was performed using the UMAP method. All cells were classified into 17 highly distinct subpopulations. The FindAllMarkers function was used to identify marker genes for each cluster. To assign cell identities and lineages, the differentially expressed genes (DEGs) in each cluster were compared to previously reported cell type marker sets.^[^ ^10^ ^]^

### Enrichment Analysis

Gene ontology (GO) analysis is a popular large‐scale functional enrichment method, encompassing biological processes (BP), cellular components (CC), and molecular functions (MF). Kyoto encyclopedia of genes and genomes (KEGG) is a globally accessible database that stores fundamental data on genomes, biological pathways, diseases, and drugs. The clusterProfiler software package^[^ ^11^ ^]^ was used for KEGG and GO pathway analyses.

### Identifying Crosstalk Between Cells in UC

To further investigate the role of colonic epithelial cells in the pathogenesis of UC, the CellChat^[^ ^12^ ^]^ package to evaluate ligand‐receptor interactions between immune and non‐immune cell components in colonic tissues was used.

### High‐dimensional Weighted Gene Co‐expression Network Analysis (hdWGCNA)

HdWGCNA^[^ ^13^ ^]^ was performed to identify key genes associated with colonic epithelial cells in metastatic samples. The “PickSoftThreshold” function was used to determine the soft‐thresholding power (β) for network construction and selected a threshold of 8. The similarity matrix based on transcript expression levels was transformed into an adjacency matrix, which was then used to generate a topological overlap matrix (TOM) to estimate network connectivity. Based on differences in the TOM and hierarchical clustering, genes were grouped into distinct clusters using weighted correlation coefficients. Genes with similar expression patterns were grouped into the same module. Using this approach, a total of nine gene modules were identified. The module eigengene (ME), representing the first principal component of each gene module, was considered the primary representative of each module.

### Gene Set Score

Gene set variation analysis (GSVA), AUCell, and AddModuleScore were applied to evaluate the score of 15 cell death modes‐related gene sets^[^ ^14^ ^]^ in colonic epithelial cells. AUCell^[^ ^15^ ^]^ calculates whether a critical subset of the input gene set is enriched among the expressed genes in each cell by computing the area under the curve.

### Western Blot

Proteins were extracted from cells using the RIPA method, and protein concentrations were measured using the BCA assay. Proteins were boiled at 100 °C for 5–10 min. SDS‐PAGE gels were used for protein electrophoresis, and proteins were transferred onto PVDF membranes. The membranes were blocked with 5% skim milk at room temperature for 1 h, followed by incubation with primary antibodies at 4°C overnight. The next day, membranes were washed with TBST and incubated with secondary antibodies at room temperature for half an hour. After washing with TBST, protein bands were visualized using a chemiluminescence instrument (Bio‐rad, USA).

### Crypt Isolation

A segment of the colon, ≈1–2 cm in length, was identified and excised. The excised tissue was then opened, rinsed with cold phosphate‐buffered saline, and placed in a calcium chelation solution at room temperature for 30 min. The chelation solution was composed of 966 mmol/L NaCl, 1.5 mmol L^−1^ KCl, 10 mmol L^−1^ HEPES, 10 mmol L^−1^ Tris, 27 mmol L^−1^ Na‐EDTA or Na‐EGTA, 45 mmol L^−1^ sorbitol, 28 mmol L^−1^ sucrose, and 0.1% bovine serum albumin. After chelation, the tissue was manually agitated to facilitate crypt release. The resulting solution was then centrifuged at 500×g for 2 min, and the isolated crypts were collected.^[^ ^16^ ^]^

### **RT‐**qPCR

Total RNA was extracted from samples using the Universal Total RNA Extraction Kit (Accurate Biology, China) following the manufacturer's instructions. First‐strand cDNA was synthesized using the PrimeScript RT Reagent Kit (Takara, Japan). Primer pair sequences can be found in Table S1 (Supporting Information). The relative quantification method 2^−∆∆Ct^ was used to measure gene expression levels.

### Immunofluorescence

Paraffin‐embedded tissues were evaluated using immunofluorescence assays. In brief, tissue sections of 5 µm thickness were subjected to deparaffinization and rehydration processes. Subsequently, antigen retrieval was performed using citrate buffer with a microwave method. After cooling, samples were treated with a 3% hydrogen peroxide solution at room temperature for 10 min to inhibit endogenous peroxidase activity. Next, slides were blocked with a 5% BSA solution at room temperature for 1 h. Following this, the slides were incubated with the following primary antibodies at 4 °C overnight. After washing with PBS, the sections were incubated with Alexa Fluor‐conjugated secondary antibodies at room temperature for 1 h. After multiple washes with PBS, tissue sections were stained with DAPI and imaged under a microscope.

### Co‐Immunoprecipitation (Co‐IP)

Cell lysates were incubated with poly(rC) binding protein 1 (PCBP1) monoclonal antibody (Abcam, ab168377), ALR polyclonal antibody (11293‐1‐AP, Proteintech), and IgG (30000‐0‐AP, Proteintech) at 4 °C overnight, followed by incubation with protein A/G magnetic beads for 4 h. Subsequently, the complexes were pelleted and washed with PBST buffer. The bound proteins were then mixed with an equal volume of 1× loading buffer and heated at 95 °C for 5 min. The samples were subjected to western blot or tandem mass spectrometry (MS) analysis performed by Fitgene Biotechnology Co., Ltd. (China).

### Correlation Analysis

Spearman's method was used for correlation analysis between single‐cell transcriptomic data and bulk transcriptomic data.

### Ferroptosis‐Related Mitochondrial Genes (FRMGs)

FRMGs were summarized from PubMed (https://pubmed.ncbi.nlm.nih.gov/) and GENECARD databases (https://www.genecards.org) between January 2022 and December 2024, identifying a total of 121 ferroptosis‐related mitochondrial genes.

### Animal and Animal Models

*Gfer* ^flox/flox^mice (CON) were a donation from Professor Xiaohui Liao of the Second Affiliated Hospital of Chongqing Medical University. *Vil1*‐MerCreMer mice were purchased from Saiye Biotechnology (Jiangsu, China). C57 wild‐type mice (WT) were purchased from the Laboratory Animal Center of Chongqing Medical University. To generate Intestinal epithelium‐specific *Gfer* knockout mice, hemizygous *Vil1*‐MerCreMer transgenic mice were first crossed with *Gfer* ^flox/flox^ mice, and *Gfer* ^flox/−^, *Vil1*‐MerCreMer offspring were then crossed with *Gfer* ^flox/flox^ mice to generate mice with the following genotypes: *Gfer* ^flox/flox^, *Vil1*‐MerCreMer (*Gfer* CKO). The mice were developed on a mixture of C57BL/6 genetic background. At 6 weeks of age, mice were intraperitoneally injected with tamoxifen at a dose of 40 mg kg^−1^, administered once every 24 h for five consecutive days. After the final injection, the mice were isolated for 24 h before being returned to the standard animal housing facility. At 8 weeks of age, CON and *Gfer* CKO mice were given 3.0% dextran sulfate sodium (DSS) in their drinking water for 7 days to establish an acute colitis mouse model. Wild‐type mice receiving DSS were intraperitoneally injected with MitoBloCK‐6 on the third day at a dose of 10 mg kg^−1^ per mouse, with injections repeated daily for 5 days. During the experiment, the mice's body weight was recorded, and each mouse was scored for the severity of colitis, considering stool consistency, the presence of blood in stool, and weight loss to calculate the disease activity index (DAI) for each mouse every day. The DAI assessment for colitis, based on a 0–4 scoring system assigned to the parameters, with the maximum score of 12, was performed as previously proposed by Kihara et al.^[^ ^17^ ^]^ Throughout the acclimation and study period, all animals were maintained under specific pathogen‐free conditions at a temperature of 21 ± 2 °C and relative humidity of 45% ± 10%, with a 12 h light/dark cycle. All animal experimental protocols were conducted in accordance with the NIH Guide for the Care and Use of Laboratory Animals and were approved by the Chongqing Medical University Institutional Animal Care and Use Committee.

### Genotyping Identification

DNA was extracted from the tail, followed by PCR amplification using *Vil1*‐MerCreMer and *Gfer* ^flox/flox^ primers. The resulting PCR products were analyzed via agarose gel electrophoresis. Mice in the *Gfer* CKO group exhibited a positive band at 450 bp for the *Gfer* ^flox/flox^‐related primers and an additional positive band at 347 bp for the *Vil1*‐MerCreMer primers. Representative genetic identification images for both the *Gfer* CKO and CON groups were provided, with molecular weight markers labeled in base pairs.

### Clinical Samples

Clinical samples were obtained from the Endoscopy Center of the Department of Gastroenterology, the Second Affiliated Hospital of Chongqing Medical University. Endoscopic tissue samples from UC patients were collected during endoscopic examinations, including samples from the lesion sites as well as adjacent, relatively normal tissues (Normal Control, NC). The diagnosis of UC was based on clinical symptoms, endoscopic and radiological findings, and histological evidence. This study was approved by the Ethics Committee of the Second Affiliated Hospital of Chongqing Medical University.

### Hematoxylin and Eosin (H&E) Stain

The paraffin‐embedded colon tissue was sectioned at a thickness of 5 µm, stained with H&E (SevierBio, Wuhan, China), and subsequently observed under a microscope.

### Alcian Blue and Periodic Acid‐Schiff (PAS) Stain

The paraffin‐embedded colon tissue was sectioned at a thickness of 5 µm, stained with Alcian blue and PAS (SevierBio, Wuhan, China), and subsequently observed under a microscope.

### Cell Culture

The FHC colon epithelial cell line was used, FHC cells were purchased from the American Type Culture Collection (#CRL‐1831, ATCC, Manassas, VA, USA).^[^ ^18^ ^]^ An in vitro model of LPS‐induced colitis was performed on FHC colonic epithelial cells. FHC cells were incubated in DMEM medium (Gibco, Grand, USA) with 10% fetal bovine serum (Gibco, Grand) and 1% penicillin–streptomycin (Gibco, Grand, USA) at 37 °C and 5% CO_2_. Lipopolysaccharide (LPS) (#L2880, Sigma‐Aldrich, St. Louis, MO, USA) was suspended in DMEM to a final concentration of 100 µg mL^−1^.

### Lentiviral Transfection

FHC cells were transfected with *GFER* overexpressing lentivirus (Genechem, China) construct FHC cells (*GFER* OE), total RNA and total protein were extracted, and mRNA and protein expression were measured by PCR and western blot, respectively.

### Measurement of Lipid Peroxidation (MDA) and Glutathione (GSH)

Intracellular MDA and GSH levels were measured using an MDA assay kit (Elabscience Biotechnology, Wuhan, China) and a reduced GSH assay kit (Beyotime, Shanghai, China) respectively, according to the manufacturer's instructions.

### Measurement of Ferrous Ion Levels

Intracellular and tissue ferrous ion levels were measured using a ferrous ion assay kit (Elabscience Biotechnology, Wuhan, China) following the manufacturer's protocol.

### Molecular Docking

The crystal structures of the target proteins PCBP1 and GFER were downloaded from the Protein Data Bank (https://www.rcsb.org/) and processed using AutoDock Vina(https://vina.scripps.edu/).

### Measurement of ROS

ROS levels in cells were assessed using the fluorescent probe DCFH‐DA (Beyotime, Shanghai, China). After removing the cell culture medium, cells were washed twice with PBS and incubated with DCFH‐DA (10 µm) at 37 °C for 30 min. Images were captured under a microscope.

### Superoxide Anion Detection

Superoxide anion levels in cells were detected using dihydroethidium (DHE) staining (Beyotime, Shanghai, China). Cells were washed twice with PBS after removing the culture medium and incubated with DHE (5 µM) at 37 °C for 30 min. Images were taken under a microscope.

### **Gene** Interference

Small interfering RNAs (siRNAs) targeting *PCBP1*, peroxisome proliferator‐activated receptor gamma (*PPAR‐γ*), peroxisome proliferator‐activated receptor‐γ coactivator‐1α (*PGC‐1α*), along with their scrambled controls, were purchased from Sangon Biotech (Shanghai, China). Transfection of the respective siRNA was performed using lipofectamine 3000 (Thermo Fisher Scientific, Massachusetts, USA) according to the manufacturer's protocol.

### Transmission Electron Microscopy (TEM)

Samples were immediately fixed in electron microscopy fixative at 4 °C for 2–4 h. Sample processing and identification were performed by Wuhan Servicebio Technology Co., Ltd. (Wuhan, China). Figures were obtained using a transmission electron microscope.

### Chromatin Immunoprecipitation (ChIP) Assay

The ChIP assay was performed using a ChIP kit (Baixin Biotechnology, China). FHC cells were incubated with or without LPS, and cell samples were collected to enrich chromatin fragments bound to PPAR‐γ using the ChIP method. According to the manufacturer's protocol, chromatin was immunoprecipitated overnight at 4 °C using an anti‐PPAR‐γ antibody or a negative control IgG antibody. DNA samples were purified with magnetic beads, amplified using primers, and analyzed on an agarose gel with GelRed nucleic acid gel stain. Figures were captured using a Bio‐Rad gel imaging system.

### Dual‐Luciferase Reporter Assay

FHC cells were transfected with *PPAR‐γ* plasmids and either wild‐type glutathione peroxidase 4 (*GPX4*‐wt) or mutant *GPX4* (*GPX4*‐mut) plasmids, which were obtained from GeneCreate Biotechnology (Wuhan, China). 48 h after transfection, the dual‐luciferase reporter assay was performed according to the instructions of the Dual‐Luciferase Reporter Assay Kit (GeneCreate, Wuhan, China).

### Statistical Analysis

Statistical analyses were conducted using R software (version 4.4.1) and Prism software (version 8.0). Comparisons between two groups with normally distributed variables were made using an unpaired Student's *t*‐test. For comparisons among three or more groups, one‐way ANOVA was first used to determine significant differences among groups, followed by Tukey's multiple comparison test to identify specific group differences. A p < 0.05 was considered statistically significant. In this study, all in vivo and in vitro experiments were performed with no less than three independent biological replications (*n* ≥ 3) to ensure the reproducibility and statistical reliability of the results.

### Ethics Approval and Consent to Participate

Informed consent was obtained from each patient before the endoscopic examination, and the project was approved by the Medical Ethical Committee of the Second Affiliated Hospital of Chongqing Medical University (approval no. 49). All animal experiments were ethically approved by the Chongqing Medical University Institutional Animal Care and Use Committee (approval no. IACUC‐CQMU‐2024‐0088).

## Conflict of Interest

The authors declare no conflict of interest.

## Author Contributions

Y.S. and F.T. are co‐first authors. Y.S. contributed to visualization, validation, methodology, investigation and conceptualization, wrote the original draft and wrote, reviewed and edited the final manuscript. F.T. contributed to visualization, methodology, investigation, conceptualization, and formal analysis. Q.S. contributed to validation and investigation and wrote, reviewed and edited the final manuscript. X.L. and Z.M. supervised the project, acquired resources and funding. L.L. supervised the project, acquired resources and funding, and wrote, reviewed and edited the final manuscript.

## Supporting information



Supporting Information

Supplemental Table 1

## Data Availability

The data that support the findings of this study are available from the corresponding author upon reasonable request.
