# Supplementary material for: Single‐Cell Transcriptomics Unravels Growth Factor Erv1‐Like Mediated Ferroptosis as a Key Driver of Intestinal Epithelial Dysfunction in Ulcerative Colitis
Source: Adv Sci (Weinh). 2025 Oct 16;12(45):e02014. doi: 10.1002/advs.202502014 (PMC12677609; doi:10.1002/advs.202502014)
Supplement: Supplementary file 1 — Supporting Information [file ADVS-12-e02014-s002.docx]

**Supplementary Material**

**Supplementary Materials and Methods**

**Longitudinal Single-Cell Sequencing Data Analysis**
To assess the association of *GFER* expression levels with disease severity, treatment response (pre-treatment and post-treatment), disease versus control groups, and inflammatory tissue subtypes, and thereby provide clearer insights into the potential translational implications for UC, we analyzed longitudinal single-cell RNA sequencing data from the GSE282122 dataset. This publicly available dataset includes biologically untreated samples from patients with UC (n=22), Crohn’s disease (CD, n=16), and healthy controls, featuring longitudinal samples collected before and after treatment, along with detailed clinical metadata. The analytical methods and R packages used were consistent with those described in the previous Methods section.

**Analysis of Cell Subpopulation Proportions Using Linear Mixed-Effects Models**

To evaluate systematic differences in the proportions of cellular subpopulations between healthy controls and UC patients, while controlling for potential confounding factors, we constructed linear mixed-effects models (LMMs) based on the relative proportions of each cell subpopulation at the sample level. Model construction and fitting were performed using the **lme4** package in R.

**AAV Injection**
To induce overexpression of Gfer in the intestine, mice were intraperitoneally injected with AAVs at a dose of 10¹¹ viral genomes per mouse, administered 7 days prior to UC induction. DSS was dissolved in distilled water to a final concentration of 3.0%. Mice in the colitis group were given free access to the DSS solution for 7 days, with the solution being replaced every other day. Mice were divided into four groups (n = 10 mice per group): (1) Negative control group (AAV-NC), (2) Gfer overexpression group (AAV-*Gfer*), (3) DSS + negative control group (AAV-NC+DSS), (4) DSS + Gfer overexpression group (AAV-*Gfer*+DSS).

**Supplementary Figures**

**Figure S1**

**
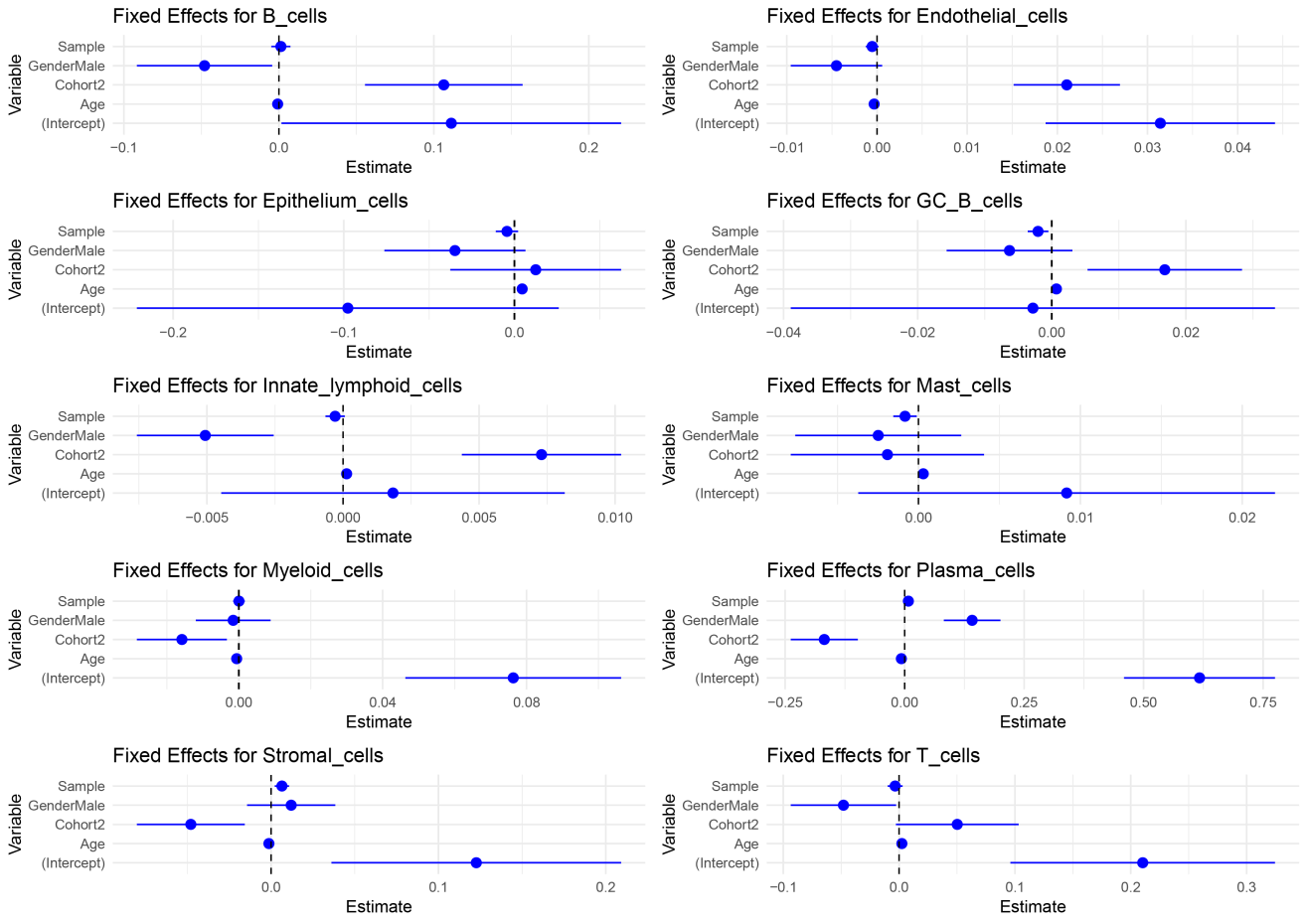
**

**Exploring the Impact of Sex, Age, and Cohort on Cell Type Proportions Using a Mixed-Effects Model**

This figure presents the results of a linear mixed-effects model used to investigate the influence of sex, age, and cohort on the proportions of various cell types. Specifically, it displays the estimated coefficients and confidence intervals for the fixed effects in the model. Each panel represents a distinct cell subpopulation. For each cell type, the plot illustrates the direction, magnitude, and statistical significance of the estimated effects for variables such as cohort, sex, and age.

**Figure S2**


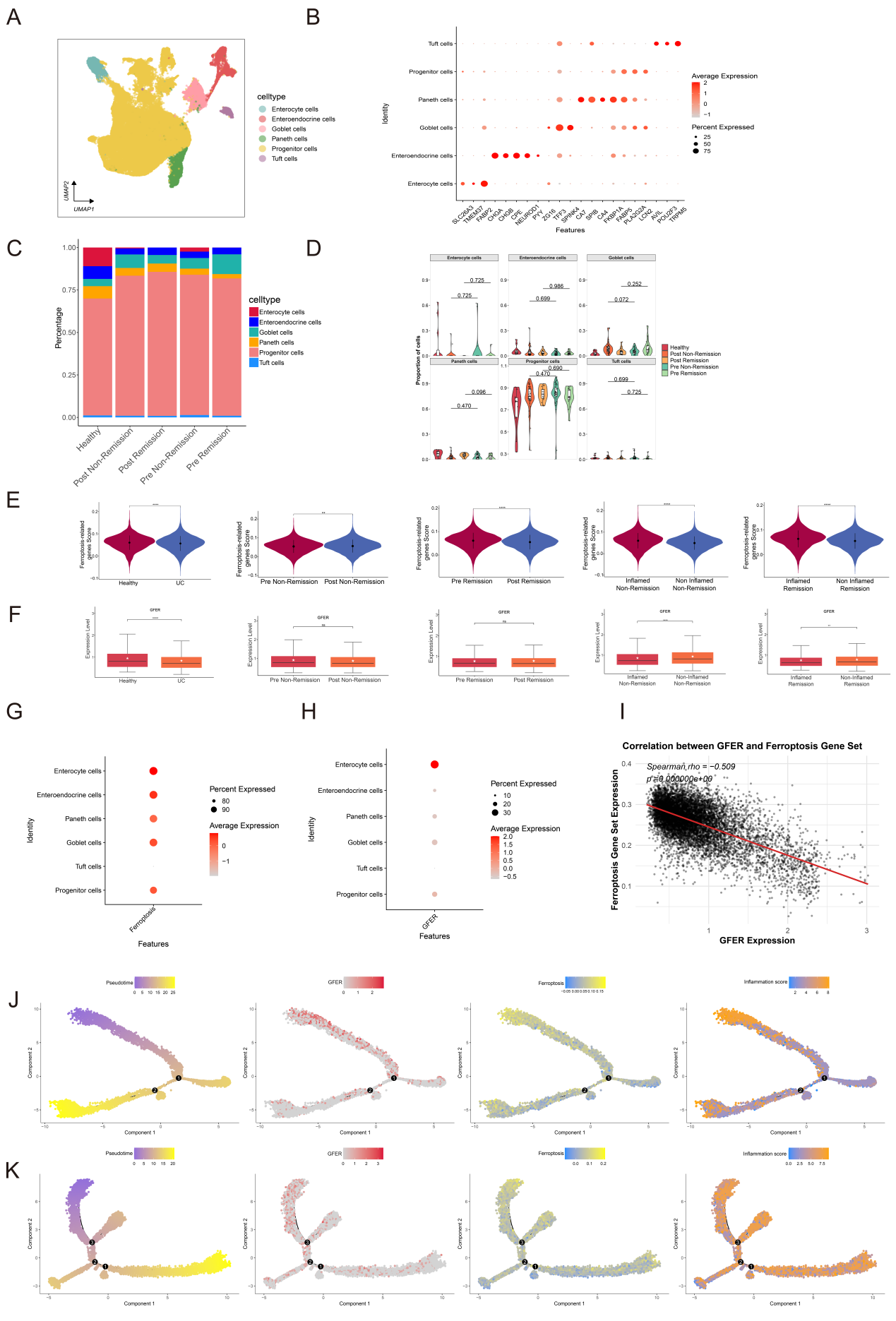


**Longitudinal Single-Cell Sequencing Data Analysis**

(A). UMAP plot displaying epithelial cell subpopulations.

(B). The dot plot showing marker gene expression for the epithelial cell subpopulations.

(C). The bar plot shows the changes in the proportion of cell types across different groups.

(D). The violin plot displays the changes in cell numbers across different groups.

(E).The violin plot compares ferroptosis scores under different pathological states.

(F). The box plot compares *GFER* expression levels under different pathological states.

(G). The dot plot illustrates the distribution of ferroptosis scores among different cell types, with enterocyte cells showing the highest enrichment.

(H). The dot plot displays the expression distribution of the *GFER* gene among different cell types, with enterocytes exhibiting the highest expression level and the largest proportion of cells expressing the gene.

(I). The scatter plot shows a negative correlation between *GFER* expression and the ferroptosis gene set enrichment score (Spearman’s ρ = –0.509, P < 2.2e–16).

(J). The single-cell pseudotime trajectory plot (Monocle) shows the dynamic expression changes of the ferroptosis pathway, *GFER*, the ferroptosis gene set, and the inflammation pathway during different developmental stages in UC remission.

(K). The single-cell pseudotime trajectory plot (Monocle) shows the dynamic expression changes of the ferroptosis pathway, *GFER*, the ferroptosis gene set, and the inflammation pathway during different developmental stages in UC non-remission.

**Figure S3**

**
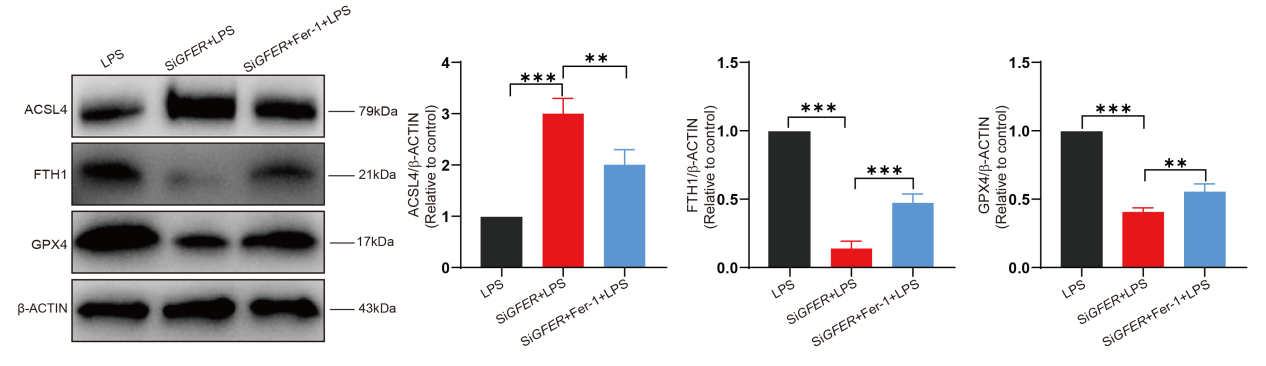
**

In LPS-induced cellular inflammation, silencing of *GFER* (Si*GFER*) was followed by treatment with the ferroptosis inhibitor Ferrostatin-1. Western blotting was used to assess the expression levels of ferroptosis-related markers such as ACSL4, FTH1, and GPX4, suggesting that GFER exerts its effect by inhibiting ferroptosis.

**Figure S4**

**
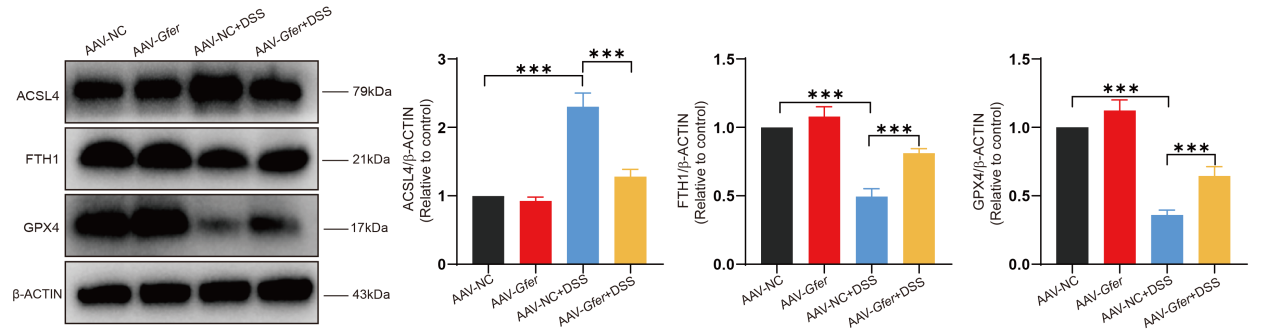
**

In vivo, overexpression of *Gfer* in the mouse intestine was validated via western blotting by examining ACSL4, FTH1, and GPX4 expression, indicating that *Gfer* overexpression suppresses ferroptosis in mice with colitis.

**Figure S5**

**
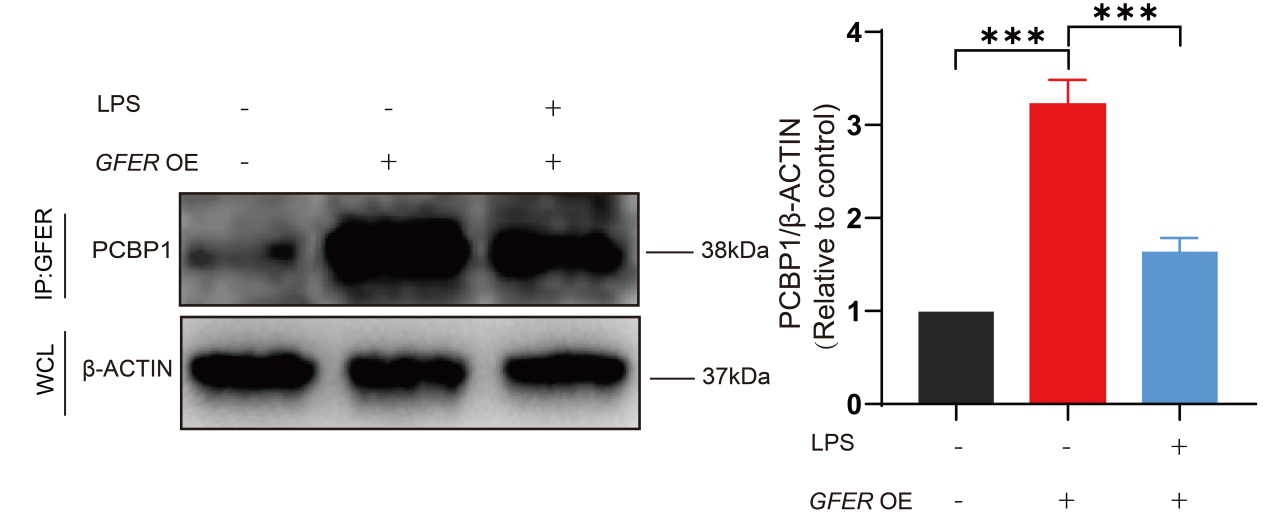
**

Co-immunoprecipitation was performed to examine the effect of *GFER* overexpression on its interaction with PCBP1. Overexpression of *GFER* enhanced its binding to PCBP1, while LPS stimulation suppressed the interaction between GFER and PCBP1.

**Figure S6**

**
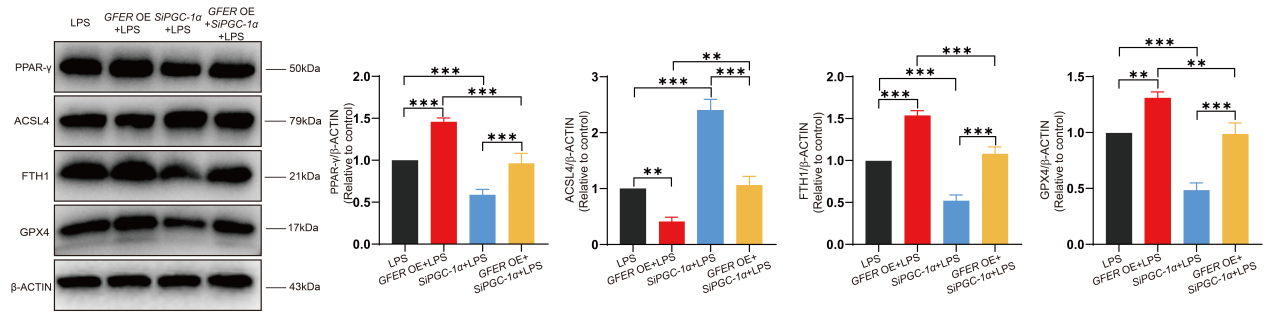
**

In FHC cells, simultaneous *GFER* overexpression and *PGC-1α* silencing (Si*PGC-1α*) were performed. Western blot analysis of PPAR-γ, ACSL4, FTH1, and GPX4 revealed that Si*PGC-1α* partially reversed the inhibitory effect of GFER on ferroptosis in LPS-induced colonic epithelial inflammation.
