# Supplementary material for: Single‐Cell Transcriptomics Unravels Growth Factor Erv1‐Like Mediated Ferroptosis as a Key Driver of Intestinal Epithelial Dysfunction in Ulcerative Colitis
Source: Adv Sci (Weinh). 2025 Oct 16;12(45):e02014. doi: 10.1002/advs.202502014 (PMC12677609; doi:10.1002/advs.202502014)
Supplement: Supplementary file 2 — Supplemental Table 1 [file ADVS-12-e02014-s001.docx]

**Table S1 Primer sequences**

|  |  |  |  |
| --- | --- | --- | --- |
| gene | species |  | primer sequences |
|  |  |  |  |
|  |  |  |  |
| *Gfer*^flox/flox^ | mouse | Forward | 5'-GCTCAAGCCAATCTCTCAGCTT-3' |
|  |  | Reverse | 5'-ATCTAGAGTTTCTAGGTAGGGCCTG-3' |
|  |  |  |  |
| *Vil1*-MerCreMer | mouse | Forward | 5'-CTCTACTGGAGGAGGACAAACTG-3' |
|  |  | Reverse | 5'-AATTGGTAGGTAGTTTCACACCCC-3' |
|  |  |  |  |
| *Gfer* | mouse | Forward | 5'-GCGCAAGGCATAGAGACGA-3' |
|  |  | Reverse | 5'-GTCCGCATCCACGACTTGAA-3' |
|  |  |  |  |
| *Acsl4* | mouse | Forward | 5'-CCTGAGGGGCTTGAAATTCAC-3' |
|  |  | Reverse | 5'-GTTGGTCTACTTGGAGGAACG-3' |
|  |  |  |  |
| *Gpx4* | mouse | Forward | 5'-GATGGAGCCCATTCCTGAACC-3' |
|  |  | Reverse | 5'-CCCTGTACTTATCCAGGCAGA-3' |
|  |  |  |  |
| *Fth1* | mouse | Forward | 5'-CAAGTGCGCCAGAACTACCA-3' |
|  |  | Reverse | 5'-GCCACATCATCTCGGTCAAAA-3' |
|  |  |  |  |
| *Ppar-γ* | mouse | Forward | 5'-AAGAAGCGGTGAACCACTGA-3' |
|  |  | Reverse | 5'-GGAATGCGAGTGGTCTTCCA-3' |
|  |  |  |  |
| *Pgc-1α* | mouse | Forward | 5'-TGTCGGAAGACACCTTCCTCT-3' |
|  |  | Reverse | 5'-AGCAGCACACTGGTTGGAAG-3' |
|  |  |  |  |
| β-Actin | mouse | Forward | 5'-GCAGGAGTACGATGAGTCCG-3' |
|  |  | Reverse | 5'-ACGCAGCTCAGTAACAGTCC-3' |
|  |  |  |  |
| *FTH1* | human | Forward | 5'-TCCTACGTTTACCTGTCCATGT-3' |
|  |  | Reverse | 5'-GTTTGTGCAGTTCCAGTAGTGA-3' |
|  |  |  |  |
| *GPX4* | human | Forward | 5'-GAGGCAAGACCGAAGTAAACTAC-3' |
|  |  | Reverse | 5'-CCGAACTGGTTACACGGGAA-3' |
|  |  |  |  |
| *ACSL4* | human | Forward | 5'-CATCCCTGGAGCAGATACTCT-3' |
|  |  | Reverse | 5'-TCACTTAGGATTTCCCTGGTCC-3' |
|  |  |  |  |
| *GFER* | human | Forward | 5'-CAGAAGCGGGACACCAAGTTT-3' |
|  |  | Reverse | 5'-CACACTCCTCACAGGGGTAA-3' |
|  |  |  |  |
| *PPAR-γ* | human | Forward | 5'-TCGAGGACACCGGAGAGG-3' |
|  |  | Reverse | 5'-CACGGAGCTGATCCCAAAGT-3' |
|  |  |  |  |
| *PGC-1α* | human | Forward | 5'-TGCATGAGTGTGTGCTCTGT-3' |
|  |  | Reverse | 5'-GCACACTCGATGTCACTCCA-3' |
|  |  |  |  |
| β-ACTIN | human | Forward | 5'-GCCTCGCCTTTGCCGAT-3' |
|  |  | Reverse | 5'-AGGTAGTCAGTCAGGTCCCG-3' |
